# Maternal Consumption of Milk or Dairy Products During Pregnancy and Birth Outcomes: A Systematic Review and Dose-Response Meta-Analysis

**DOI:** 10.3389/fnut.2022.900529

**Published:** 2022-06-09

**Authors:** Donghui Huang, Qijun Wu, Xin Xu, Chao Ji, Yang Xia, Zhiying Zhao, Huixu Dai, Hang Li, Shanyan Gao, Qing Chang, Yuhong Zhao

**Affiliations:** ^1^Department of Clinical Epidemiology, Shengjing Hospital of China Medical University, Shenyang, China; ^2^Clinical Research Center, Shengjing Hospital of China Medical University, Shenyang, China; ^3^Key Laboratory of Precision Medical Research on Major Chronic Disease, Liaoning, China

**Keywords:** pregnant woman, milk, dairy, fetal growth, birth outcome, dose-response meta-analysis

## Abstract

**Purpose:**

This study aimed to systematically review current evidence and quantitatively evaluate the associations between milk or dairy consumption during pregnancy and birth outcomes.

**Methods:**

This systematic review had been reported in accordance with the guidelines of Preferred Reporting Items for Systematic Reviews and Meta-Analyses (PRISMA) statement. A supplementary literature search in PubMed, Web of Science, Cochrane Library, and Embase was conducted on 30 March 2021. Studies that assessed the association of maternal consumption of milk or dairy with birth-related outcomes were identified. The dose-response meta-analyses of continuous data and categorical data were applied. One-stage approach and two-stage approach were used where appropriate.

**Results:**

In total, 42 studies were eligible for the present systematic review, and 18 of them were included in the outcome-specific meta-analyses. The dose-response meta-analysis [Number of studies (*N*) = 9] predicted a maximum mean change in birthweight of 63.38 g [95% Confidence Interval (CI) = 0.08, 126.67] at 5.00 servings per day. Intake of dairy products had the greatest protective effect on small for gestational age at a maximum of 7.2 servings per day [Relative risk (RR) = 0.69, 95% CI = 0.56, 0.85] (*N* = 7). The risk of large for gestational age was predicted to be maximum at 7.20 servings per day of dairy consumption, with the RR and 95% CI of 1.30 (1.15, 1.46; *N* = 4). In addition, the relationship between dairy consumption and low birth weight (RR = 0.70, 95% CI = 0.33, 1.50; *N* = 5) and pre-mature birth (RR = 1.13, 95% CI = 0.87, 1.47; *N* = 5) was not significant, respectively.

**Conclusions:**

Maternal consumption of dairy during pregnancy has a potential effect on fetal growth. Further well-designed studies are warranted to clarify the specific roles of individual dairy products.

**Systematic Review Registration:**

identifier: PROSPERO 2020 CRD42020150608

## Introduction

Adverse birth outcome is a global health issue. According to the Global Burden of Disease Study, pre-mature birth (PB) is one of ten leading causes of total years of life lost, and one of three leading global causes of death in children under-5 years (1). It is estimated that PB is responsible for 0.943 million neonatal deaths and 1.055 million under-five deaths per year (2). Besides, those pre-term survivors are also likely to suffer lifelong challenges from physical health (3), neurological development (4), and psychosocial deficits (5). Similarly, other adverse birth outcomes also result in lasting adverse consequences. As evidence suggests, birth characteristics have been linked to children's cardiometabolic risk (6), cancer prognosis (7), and age-related cognitive dysfunction (8). Furthermore, birth defects, the most serious of all adverse birth outcomes, increase the risk of cancer persisted into adulthood (9), and there remains a gradual decline in survival beyond 1 year of age that exceeded that of the general population (10).

In recent years, especially since the inception of the “developmental origins of health and disease” theory, the impact of maternal nutrition has been increasingly emphasized (11). Mounting evidence has indicated that maternal diet during pregnancy, a modifiable factor, is associated with birth outcomes (12, 13). Among these nutritious foods during pregnancy, milk and its derivatives receives certain attention (14). There are increasing reports of maternal dairy intake contributing to birth outcomes; however, some associations remain inconclusive with the growing body of evidence (15, 16).

Although a published systematic review and meta-analysis has assessed the effect of consuming dairy products over perinatal outcomes, the dose-response relationships are still unclear (17). In addition, the birth outcomes range widely from birth anthropometry, birth defects to other adverse birth events, these extensive outcomes need to be further summarized (16, 17). Therefore, the present study aimed to systematically review current evidence covering all birth outcomes, and quantitatively evaluate the dose-response associations of milk or dairy consumption during pregnancy with birth outcomes.

## Methods

This systematic review had been reported in accordance with the guidelines of Preferred Reporting Items for Systematic Reviews and Meta-Analyses (PRISMA) statement. This systematic review was registered in the International Prospective Register of Systematic Reviews (PROSPERO) as CRD42020150608.

### Search Strategy

The first systematic literature search in PubMed, Web of Science, Cochrane Library, and Embase was conducted on May 23, 2019, and the supplementary search was performed on 30 March 2021. The search strategy was predefined according to the “PICO” principle: “P”-pregnant woman, “I”-milk or dairy consumption, “C”-no or low consumption of milk or dairy, “O”-birth outcomes. The complete search strategies are presented in Supplementary Material 1. In addition, there were no restrictions on publication date or language. Two authors (Huang DH and Ji C) independently conducted the literature search. Disagreements were resolved by discussion with a third investigator (Chang Q).

### Study Selection: Inclusion and Exclusion Criteria

Retrieved references were exported to the EndNote reference manager for crude deduplication and organization. Four independent reviewers (Huang DH, Xu X, Gao SY, and Dai HX) screened the titles and abstracts of the studies for eligibility. Two reviewers (Xu X and Huang DH) further independently reviewed the full texts of potentially relevant articles based on the inclusion and exclusion criteria. Discrepancies were resolved by discussion, with another reviewer (Wu QJ) consulted as and when necessary. The studies were selected based on priori-determined eligibility criteria.

The inclusion criteria were as follows: (1) study design was interventional (dairy consumption as an intervention) or observational (cohort study, case-control study, and cross-sectional study with dairy consumption as an exposure); (2) exposure of interest was the consumption of milk and dairy products during pregnancy, including yogurt, cheese, butter, and ice cream; (3) outcomes of interest were birth-related outcomes, including birth anthropometric measurements, PB, spontaneous abortion (SA), and congenital abnormality; and (4) if the study had a different objective than the research question being addressed in the present review, it was considered eligible if the association between dairy intake during pregnancy and birth outcomes was adequately described (information on the above three criteria was required).

The exclusion criteria were as follows: (1) study without a control group; (2) study with non-human subjects; (3) exposure time examined was not during pregnancy; (4) exposure of interest was dietary pattern, or combined diet, or fortified dairy products; (5) exposure of interest was nutrients from dairy source; (6) outcome was not at birth, including outcomes of fetuses, infants, children, and pregnancy complications; (7) study was not an original article, namely a review, conference abstract, letter, or book chapter; and (8) studies were written in languages other than English or Chinese.

### Data Extraction

The following information was extracted using a pre-designed data collection form: study characteristics (first author, year of publication, country, study design, and sample size), participant characteristics (maternal age and body mass index), exposure characteristics (types of dairy products, dietary assessment tools and whether validated, and time period covered), and outcomes characteristics (specific birth outcomes and main conclusions). Two reviewers (Li H and Huang DH) independently extracted and cross-checked the data. Inconsistencies were resolved by consensus with a third reviewer (Xia Y).

### Quality Assessment

The National Institutes of Health Study Quality Assessment Tools were used to assess the quality of the included studies. The tools contained 12–14 questions applicable to each study design and aimed to evaluate all aspects of the included studies. In case of a cohort study design, the 14 criteria used were based on the research objective, study population, exposure, outcome of interest, and statistical analysis. Each item was scored as “Yes” (+1), “No” (+0), “Cannot Determine” (+0), “Not Applicable” (+0), and “Not Reported” (+0). The sum of each item was the study quality score. The score was then summarized into three grades: a score ≥10 was graded Good, that between 5 and 9 was graded Fair, and that ≤4 was graded Poor. Two authors (Xu X and Huang DH) independently assessed study quality, and any disagreement was resolved by consensus with the third author (Wu QJ).

### Data Synthesis and Statistical Analysis

Crude estimates were calculated using given raw data where appropriate, although data synthesis preferred the most-adjusted results. Besides, if required to switch to a reference group, the *hamling* function in the “dosresmeta” package of R software was used to estimate an alternative comparison of the dose categories (18). The dose units for dairy consumption were unified to 200 ml or 200 g according to the average size of several portion sizes (19–21). The present study aimed to pool three or more sets of continuous or categorical data using dose-response meta-analyses proposed by Crippa et al. (22, 23). When the studies included in the meta-analysis all had more than two exposure groups, the two-stage method was used; when any of the included studies had fewer than three exposure groups, the one-stage method was used (23). The pooled dose-response curve was estimated by restricted cubic splines model with three knots at the 10th, 50th, and 90th percentiles of the distribution. Both restricted maximum likelihood (REML) and maximum likelihood (ML) were adopted and the better estimation method was selected based on the lowest Akaike information criterion and Bayesian information criterion values. Sensitivity analyses were performed with alternative knots. Publication bias were detected by Egger's test and Begg's test. Subgroup analyses were performed based on the presence or absence of a specific portion size. All analyses were conducted using R: A language and environment for statistical computing (version 3.6.2). A two-tailed *P* < 0.05 was considered statistically significant.

## Results

### Literature Search and Study Selection

**Figure 1:**
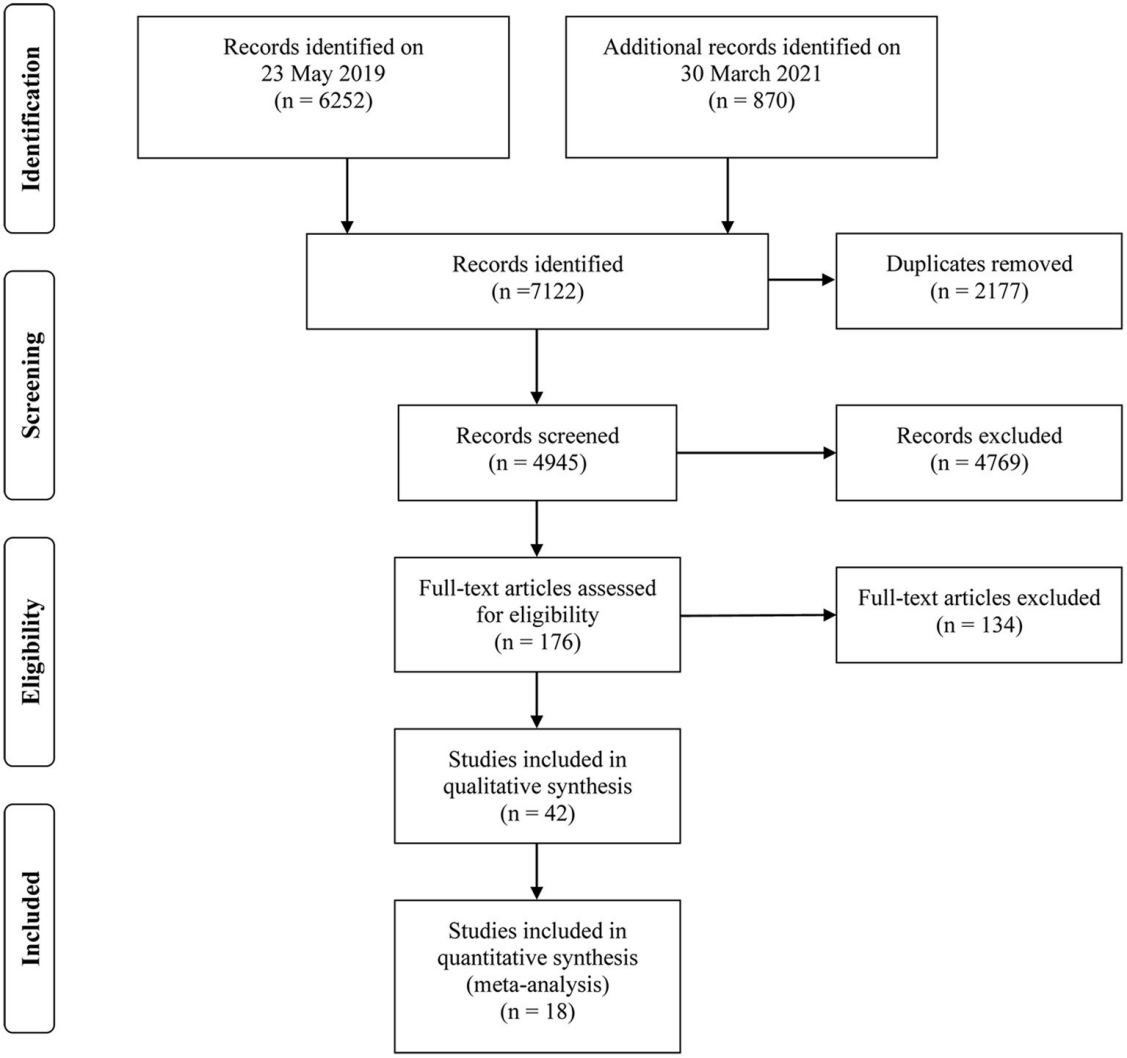
The PRISMA flow diagram of study selection.

Of 7,122 studies, 2,177 duplicates were excluded, and 4,945 studies were screened by titles and abstracts. The remaining 176 relevant studies were reviewed in full text. Precisely 134 studies were further excluded, and the reasons for exclusion are presented in Supplementary Material 2. In total, 42 studies were eligible for the present systematic review (19–21, 24–62), and 18 of them were included in the outcome-specific dose-response meta-analyses (19–21, 27–31, 33, 37, 38, 43, 44, 49, 54, 58, 61, 62). The detailed PRISMA flow diagram showing the screening process is illustrated in Figure 1.

### Study Characteristics

The detailed characteristics of the 42 included studies are shown in Table 1. There were 23 cohort studies (19–21, 26, 27, 31, 32, 36, 39, 40, 42–45, 49–52, 55, 57, 58, 61, 62), 10 cross-sectional studies (24, 28, 33, 34, 37, 47, 48, 53, 56, 59), seven case-control studies (25, 29, 35, 41, 46, 54, 60), and two interventional studies (30, 38). There were 13 studies rated as Good (19, 21, 26, 30–32, 36, 45, 49, 50, 52, 58, 61), 27 as Fair (20, 24, 25, 27–29, 33–35, 39–44, 46–48, 51, 53–57, 59, 60, 62), and two as Poor (37, 38). A summary of the quality assessment is presented in Supplementary Material 3. There were over 20 types of birth outcomes, including birth weight (BW) (19–21, 24, 26–28, 30–34, 36, 38, 39, 43, 45, 47, 48, 50, 62), birth length (BL) (19, 21, 26, 30–32, 34, 36, 38, 45, 48), head circumference (19, 21, 26, 30–32, 39, 45, 48), low birth weight (LBW) (28, 37, 38, 40, 49, 53, 55, 59, 61), PB (19, 27, 28, 40, 49, 52, 57, 61), small for gestational age (SGA) (19, 29, 31, 44, 54, 57, 58, 61), large for gestational age (LGA) (19, 31, 57, 58, 61), mid-upper arm circumference (26, 32, 39), triceps skinfold (26, 32, 39), abdominal circumference (26, 31, 48), placental weight (26, 31, 45), Apgar score (27, 38, 57), SA (25, 46), hypospadias (35, 42), subscapular skinfold (26, 39), serum 25-hydroxvitamin D (30, 56), crown heel length (39), neural tube defects (41), anencephaly (41), spina bifida (41), cryptorchidism (42), blood pressure (30), bone mineral Ca (30), fat mass (30), lean mass (30), serum Ca (30), serum total protein (30), hyperbilirubinemia (57), respiratory distress (57), hypoglycaemia (57), neonatal intensive care unit (57), composite new-born outcomes (57), congenital heart disease (60), macrosomia (61), any congenital disease (49), and adverse pregnancy outcomes (51). Outcome-specific findings of dose-response meta-analyses are described below.

**Table 1:** Characteristics of included studies.

**Study; Country**	**Design, sample size**	**Population characteristics**		**Exposure**	**Dietary assessment, time period covered**	**Birth outcome**	**Significant results**	**Quality**
		**Age**	**BMI**					
Petridou (24); Greece	CS, 368	27.4	NA	DP	Validated-FFQ; During pregnancy	BW	N	Fair
Di Cintio (25); Italy	CC, 2,681	30.5	PP: 21.5	Milk; cheese; butter	FFQ; 1st trimester	SA	Milk and cheese were inversely related to risk of SA; Butter was directly associated with the risk of SA	Fair
Rao (26); India	Cohort, 626	21.4	PP: 18.1	DP	FFQ; The preceding 3-month of 18 and 28 weeks	BW, BL, HC, MUAC, AC, TS, SSS, PW	DP consumption at 18 weeks gestation was related to BW, BL, MUAC, HC, PW	Good
Chang (27); America	Cohort, 350	15.9	PP: 23.2	DP	FFQ + 24-h DR; Covering habitual intake (at the first pre-natal visit)	BW, PB, Apgar score	N	Fair
Ludvigsson (28); Sweden	CS, 14,000	29.5	NA	DP	FFQ; During pregnancy	PB, BW, LBW	The mean BW increased with milk consumption	Fair
Mitchell (29); New Zealand	CC, 1,131	29.7	23.6	DP	FFQ; At the time of conception and in the last month of pregnancy	SGA	N	Fair
Chan (30); America	RCT, 72	16.6	25.3	DP	2-day FR + unscheduled 24-h DR; From enrollment (<20 week) to delivery	BW, BL, HC, blood pressure, bone mineral Ca, fat mass, lean mass, serum Ca, serum 25-OH D, serum total protein	The BWs of the infants in the dairy group were heavier than other groups; The infants in the dairy group had higher total body Ca than infants in the control group; The 25-OH D levels were higher in the dairy group than other groups	Good
Mannion (21); Canada	Cohort, 279	30.9	PP: 23.1	Milk	Validated Repeat 24-h telephone DR; 3 or 4 random days during pregnancy	BW, BL, HC	For each cup of milk consumed per day, BW increased 41 g on average	Good
Olsen (31); Denmark	Cohort, 50,117	29.1	PP: 23.4	DP	Validated-FFQ; Previous 4 weeks of the 25 weeks of gestation	BW, AC, PW, HC, BL, SGA, LGA	Mean BW, AC, PW, HC, and BL all showed increases across the whole range of milk intake; The odds of being SGA declined with increasing consumption of milk; The odds of having a LGA increased with exposure	Good
Kanade (32); India	Cohort, 179	27.1	PP: 22.6	DP	FFQ; 1 month prior to each visit (18 weeks and 28 weeks of gestation)	BW, BL, HC, TS, MUAC	Consumption of milk at 28th week was associated with TS	Good
Xue (33); America	CS, 34,063	26.2	PP: 21.2	Milk	Questionnaire; During pregnancy	BW	Daily consumption of each additional glass of milk was associated with an increase of 5.5 g in BW	Fair
Borazjani (34); India	CS, 156	28.0	PP: 22.8	Milk	FFQ + 24-h DR; During pregnancy	BW, BL	Daily maternal milk intake during gestational period showed positive significant contribution to BW; each 1 ml increase in milk consumption was associated with 0.73 (g) rises in offspring's BW	Fair
Heppe (19); Netherlands	Cohort, 3,405	31.4	PP: 23.2	DP	Validated-FFQ; Prior 3 months of 13.5 weeks of gestation, covering the 1st trimester of pregnancy	HC, BW, BL, PB, SGA, LGA	Maternal milk consumption was positively associated with HC and BW	Good
Maslova (20); Denmark	Cohort, 58,762	≈30.0	PP: 23.4	DP	Validated-FFQ; Previous 4 weeks of gestation week 25	BW	BW was generally higher for children whose mothers consumed more dairy products.	Fair
Christensen (35); Denmark	CC, 608	30.4	22.9	Milk; non-milk DP	Questionnaire; 1st trimester	Hypospadias	N	Fair
Hrolfsdottir (36); Denmark	Cohort, 809	29.1	PP: 21.5	DP	Validated-FFQ; Previous 3 months of gestational week 30, corresponding roughly to the 2nd trimester of pregnancy	BW, BL	Milk intake of ≥150 ml/day was associated with 0.32 higher *z*-scores for BW and 0.34 higher *z*-scores for BL	Good
Sultan Azzeh (37); Saudi Arabia	CS, 147	29.4	PP: 25.8	DP	Structured questionnaire; During pregnancy	LBW	N	Poor
Li (38); China	Parallel group design study, 2,016	26.8	PP: 22.4	Milk	Periodical records; From confirmation of pregnancy (5–7 weeks) to parturition	BW, BL, LBW, apgar score	The average BW and BL of newborns were increased by 1.9 and 0.8%, respectively, after maternal supplementation with milk; the frequency of LBW was significantly decreased by maternal supplementation with milk	Poor
Shaikh (39); Pakistan	Cohort, 100	27.4	24.9	Milk	FFQ + 24-h DR; 1st and 3rd trimester	BW, TS, HC, CHL, SSS, MUAC	A statistically significant negative association was noted between maternal milk intake in well-nourished group and SSS thickness of the newborn at birth. Similarly, consumption of milk in undernourished mothers was also found associated negatively with MUAC of the newborns	Fair
Akbari (40); Iran	Cohort, 225	NA	NA	DP	FFQ; During pregnancy	PB, LBW	Mothers of pre-mature newborns consumed lower amounts of dairy products	Fair
Wang (41); China	CC, 917	NA	PP: 71.2% underweight	Milk	Validated-FFQ; 1st trimester	NTD: Anencephaly, spina bifida	A 41–50% reduction in risk of NTDs was observed at all levels of milk consumption; a 57–83% reduction in risk of spina bifida was observed at all levels of milk consumption	Fair
Brantsaeter (42); Norway	Cohort, 35,107	29.0	PP: 23.8	Organic DP	Validated-FFQ; Since the start of pregnancy to gestational week 22, covering the first 4 months of pregnancy	Hypospadias, Cryptorchidism	N	Fair
Miyake (43); Japan	Cohort, 1,319	32.0	20.9	DP; Milk	Validated-DHQ; The preceding month (median gestational week: 17th week)	BW	Intake levels of total dairy products and milk were inversely associated with baby's BW	Fair
Olmedo-Requena (44); Spain	Cohort, 973	29.7	PP: 24.0	DP	Validated-FFQ; Until the 21th weeks, covering the first half of pregnancy	SGA	An increase by 100 g/day of dairy product intake during the first half of pregnancy was seen to decrease the risk of having an infant with SGA by 11.0%	Fair
Abreu (45); Portugal	Cohort, 98	30.1	PP: Non-overweight: 60.2%	DP; milk; yogurt; cheese	3-day FR; 1st and 2nd trimesters	BW, BL, HC, PW	Total dairy and yogurt intake in the first trimester were positively associated with HC and PW, respectively	Good
Ahmadi (46); Iran	CC, 662	27.6	24.6	DP	Validated-FFQ; In the preceding 3 months	SA	There was a significant difference between the case and control groups regarding consumed servings/day of dairy products	Fair
Yan (47); China	CS, 9,050	27.6	NA	DP	FFQ; During pregnancy	BW	N	Fair
Hjertholm (48); Malawi	CS, 132	NA	NA	DP	3-day quantified interactive 24-h DR + 4-day semiquantitative 24-h DR; 7 days between 28 weeks and 35 weeks of gestation	BW, BL, HC, AC	Each additional day of milk consumption, within the seven measurement days, was associated with a 75.3 g increase in BW	Fair
Kriss (49); Mexico	Cohort, 965	26.3	26.0	Yogurt	FFQ; In the past 3 months (mean gestational week: 20.6 weeks)	PB, LBW, any congenital disease	Non-overweight women who consumed ≥5 cups of yogurt per week were 76% less likely to deliver pre-term	Good
Mukhopadhyay (50); India	Cohort, 2,036	24.4	21.7	DP	Validated-FFQ; The preceding 3 months at each visit, covering three trimesters of pregnancy	BW	BW was positively associated with intake of milk products in the 1st trimester	Good
Zerfu (51); Ethiopia	Cohort, 374	25.1	NA	DP	4-day 24-h DR; Monthly (for a total of 4 days) from enrollment to delivery	APOs	Poor or inconsistent consumption of dairy products were independently associated with higher APO risks	Fair
Ito (52); Japan	Cohort, 77,295	30.9	PP: 21.3	Yogurt; cheese	FFQ; During pregnancy	PB	Cheese intake reduced early PB risk	Good
Abera (53); Ethiopia	CS, 358	26.9	NA	DP	Questionnaire; At 3rd trimester	LBW	Consumption of dairy product had significant association with LBW	Fair
Olmedo-Requena (54); Spain	CC, 1,036	NA	NA	DP; milk; yogurt; cheese	Validated FFQ; During pregnancy	SGA	N	Fair
Shen (55); China	Cohort, 3,172	28.9	PP: 21.7	DP	Questionnaire; Previous 7 days, during 1st trimester	LBW	Consumption of dairy product 7 days per week was a protective factor for LBW	Fair
Baki Yildirim (56); Turkey	CS, 120	28.0	NA	DP	Questionnaire; During pregnancy	Neonatal serum 25(OH)D concentration	N	Fair
Assaf-Balut (57); Spain	Cohort, 2,004	32.6	PP: 23.3	Fat-free DP	The diabetes nutrition and complications trial questionnaire; Between 8–12 and 24–28 gestational weeks	PB; LGA; SGA; Apgar 1 m <5; HB; RD; HG; NICU; Composite new-born outcomes	The higher the consumption, the lower the rates of admission to NICU	Fair
Pang (58); China	Cohort, 962	28.7	PP: 21.6	DP	3-day 24-h DR; The previous 3 days of 3 visits (8–14 wks, 24–28 weeks, and 32–36 weeks)	SGA; LGA	Compared with no dairy consumption group in the 2nd trimester of pregnancy, the risk of SGA was lower in suitable dairy consumption group; Compared with no dairy consumption group in the 3rd trimester of pregnancy, the risk of SGA was lower in insufficient dairy consumption group and suitable dairy consumption group	Good
Rodrigues (59); Brazil	CS, 99	24.9	NA	DP	Validated food consumption markers form of the food and nutrition surveillance system; Seven days before giving birth	LBW	N	Fair
Li (60); China	CC, 968	28.5	PP: 21.1	DP	Questionnaire; During the early pregnancy	CHD in offspring	Maternal excessive intakes of milk products significantly decreased the risk of CHD in offspring	Fair
Sartorelli (61); Brazil	Cohort, 733	27.7	PP: 25.9	Milk	24-h DR; During pregnancy	PB; LBW; Macrosomia; SGA; LGA	A likelihood of a lower odds of having a LBW child was found among women with daily consumption of 100 ml or more of milk	Good
Voerman (62); Netherlands	Cohort, 2,466	31.9	PP: 23.2	DP	Validated FFQ; Over the prior 3 months, thereby covering the 1st trimester of pregnancy	BW	Intakes of milk differed between milk-intake groups	Fair

*AC, abdominal circumference; APOs, adverse pregnancy outcomes; BL, birth length; BMI, body mass index; BW, birth weight; CC, case control; CHD, congenital heart disease; CHL, crown heel length; CS, cross sectional; DHQ, diet history questionnaire; DP, dairy products; DR, dietary recall; FFQ, food frequency questionnaire; FR, food record; HB, hyperbilirubinemia; HC, head circumference; HG, hypoglycaemia; LBW, low birth weight; LGA, large for gestational age; MUAC, mid-upper arm circumference; N, No; NA, not applicable; NICU, neonatal intensive care unit; NTD, neural tube defects; PB, pre-mature birth; PP, pre-pregnancy; PW, placental weight; RCT, randomized controlled trial; RD, respiratory distress; SA, spontaneous abortion; SGA, small for gestational age; SSS, subscapular skinfold; TS, triceps skinfold*.

### Birth Weight

**Figure 2:**
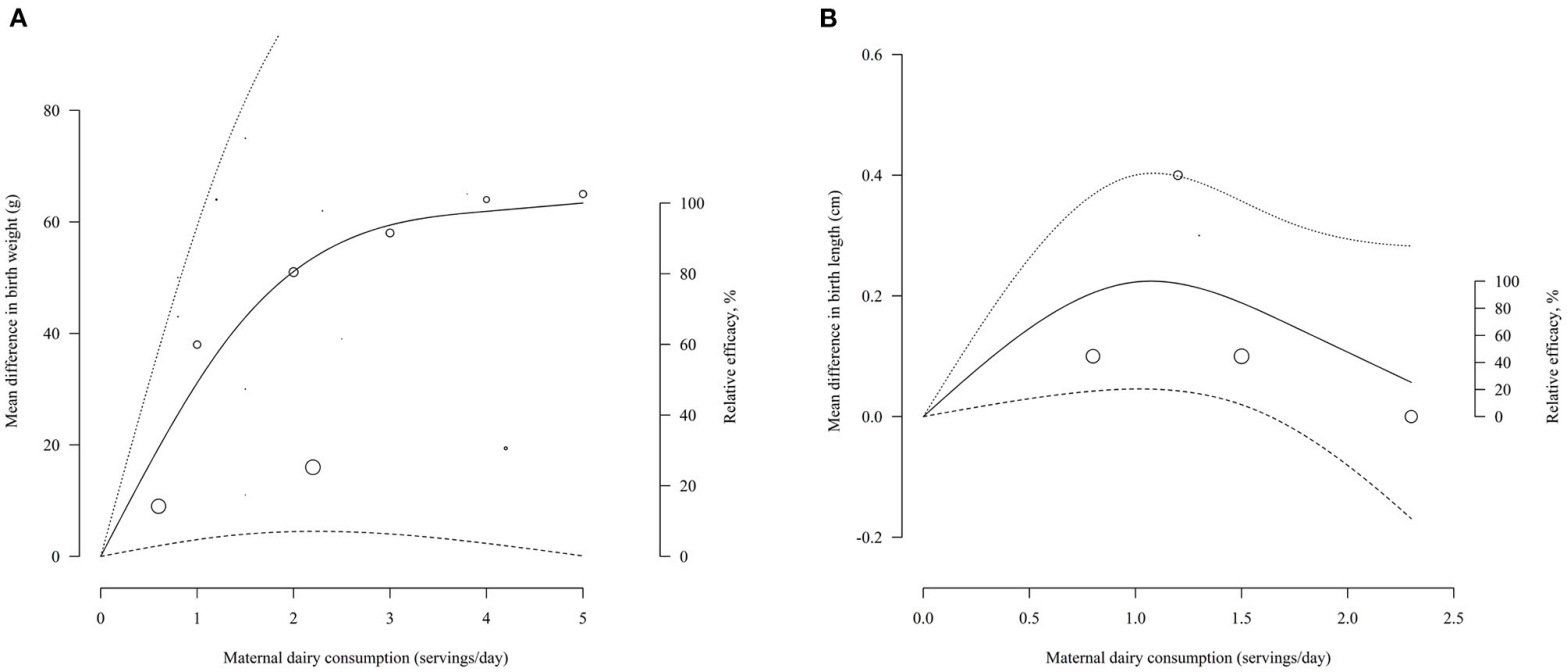
Pooled dose-response curves for mean change in birth-weight **(A)**, birth-length **(B)** with maternal dairy consumption. Dashed lines represent the 95% confidence intervals. Circles indicate observed mean differences in individual studies, and the size of circles is proportional to the precision of the mean differences. The right axis represents the percentage of the maximum predicted effect.

Nine studies were used for the one-stage dose-response meta-analysis (19–21, 27, 30, 33, 38, 43, 62). The overall pooled curve estimated by the REML method indicated a non-significant association between the mean differences in BW and increased consumption of dairy products (*P* = 0.0847) (Figure 2A). However, the first and second estimated coefficients of 33.1497 (*P* = 0.0296) and −32.4737 (*P* = 0.0263), respectively, suggested that the dose-response relationship tended to be positive. The pooled curve predicted a maximum mean change of 63.38 g (95% Confidence Interval [CI] = 0.08, 126.67) at 5.00 servings per day. The estimated dose to produce 50 and 80% of the predicted maximum effect was 1.02 and 1.97 servings/day, respectively, and the mean change in BW was 31.69 and 50.70 g, respectively. Sensitivity curves with alternative knots locations indicated the robustness of the pooled estimates (Supplementary Figure S1). Subgroup analysis showed the dose-response relationship was significant when the studies without specific portion size were excluded (*P* < 0.0001). In addition, there was no publication bias (*P-*linear = 0.3285; *P-*rank = 0.7139).

### Birth Length

Only three studies could be dose-response meta-analysis (19, 21, 38). Overall estimates fitted by the ML model suggested a marginal significant dose-response effect between dairy intake and BL (*P* = 0.0474) (Figure 2B). The first and second estimated coefficients were 0.3154 (*P* = 0.0143) and−0.2797 (*P* = 0.0306), respectively. The pooled curve predicted a maximum mean change of 0.22 cm (95% CI = 0.05, 0.40) at 1.07 servings per day. The estimated dose to produce 50 and 80% of the predicted maximum effect was 0.37 servings/day and 0.65 servings/day, respectively, and the mean change in BL was 0.11 and 0.18 cm, respectively. Sensitivity analysis with alternative knots indicated that the shapes of curves under different knots were unchanged (Supplementary Figure S2). Both Egger's test and Begg's test showed no publication bias (*P-*linear = 0.6; *P-*rank = 0.3).

### Small for Gestational Age

**Figure 3:**
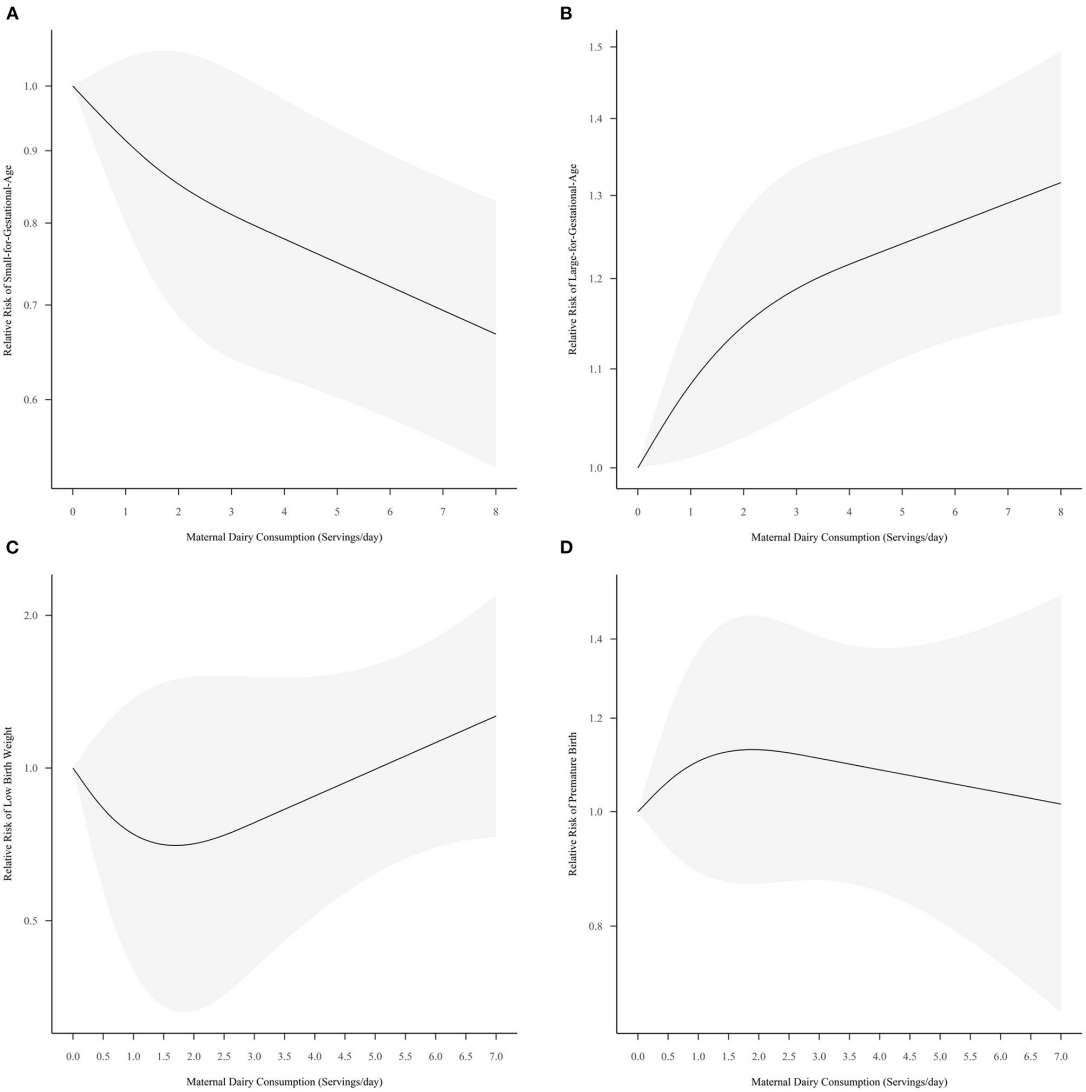
Pooled dose-response curves for the risk of small-for-gestational-age **(A)**, large-for-gestational-age **(B)**, low-birth-weight **(C)**, and pre-mature-birth **(D)** with maternal dairy consumption. The gray part in this figure represents the 95% confidence intervals.

A total of 10 sets of data from seven studies examined the dose-response relationship between dairy consumption and SGA risk (19, 29, 31, 44, 54, 58, 61). The pooled curve by ML model suggested that maternal dairy intake tended to prevent SGA (*P* = 0.0001) (Figure 3A), while the coefficients of −0.0920 (*P* = 0.2095) and 0.0449 (*P* = 0.5154) were not significant. It was predicted that the intake of dairy products had the greatest protective effect on SGA at a maximum of 7.2 servings per day [Relative risk (RR) = 0.69, 95% Confidence interval (CI) = 0.56, 0.85]. Sensitivity analysis showed that the relationship was robust (Supplementary Figure S3). The results of Egger's test and Begg's test were inconsistent, suggesting a potential publication bias (*P-*liner = 0.0409, *P-*rank = 0.7566). Subgroup analysis excluded the studies without specific portion size, and the pooled results were still significant (*P* < 0.0001).

### Large for Gestational Age

The pooled dose-response curve by six sets of data from four studies showed a significant upward trend for LGA risk with increased dairy consumption (*P* = 0.0001, Figure 3B) (19, 31, 58, 61). ML method estimated the first coefficient was 0.0870 (*P* = 0.0314), and the second coefficient was−0.1247 (*P* = 0.1662). The risk of LGA was predicted to be maximum at 7.20 servings/day of dairy consumption, with the RR and 95% CI of 1.30 (1.15, 1.46). There was a significant publication bias (*P-*liner = 0.0007, *P-*rank = 0.0169), and the sensitivity analysis showed a robust curve (Supplementary Figure S4).

### Low Birth Weight

Five studies were included in the dose-response meta-analysis (28, 37, 38, 49, 61), and the pooled results showed a non-significant relationship (*P* = 0.4451). The two coefficients estimated by ML method were −0.3968 (*P* = 0.3272) and 1.0494 (*P* = 0.2918), respectively. The dose-response curve showed a visible but not significant downward trend first and then an upward trend for LBW with increasing dairy consumption (Figure 3C). Dairy consumption showed a potential protective effect on LBW at 1.70 servings/day (RR = 0.70, 95% CI = 0.33, 1.50). The RR and 95% CI for LBW were 1.24 (0.73, 2.10) when maternal dairy consumption reached a maximum of 6.80 servings/day. Sensitivity analysis with alternative knots was shown in (Supplementary Figure S5). The publication bias was not significant (*P-*liner = 0.5609, *P-*rank = 0.3223). The pooled results were still non-significant after excluding the studies without specific portion size (*P* = 0.4646).

### Pre-mature Birth

A total of five studies were included in the one-stage dose-response meta-analysis (19, 27, 28, 49, 61). ML method estimated that this dose-response relationship was not significant (*P* = 0.6632) (Figure 3D), and the two coefficients of 0.1240 (*P* = 0.3780) and −0.2356 (*P* = 0.4107) were not statistically significant. It was predicted that the maximum risk of PB was 1.13 (95% CI: 0.87, 1.47) for 1.89 servings dairy products per day. Sensitivity analysis indicated a robust curve (Supplementary Figure S6). There was no publication bias in the included studies (*P-*liner = 0.7795, *P-*rank = 0.5858). Subgroup analysis showed the relationship was still not significant when the studies without specific portion size were excluded (*P* = 0.7439).

### Spontaneous Abortion

The meta-analyses based on three sets of data from two studies found a non-significant protective effect of dairy intake against SA (*P* = 0.0842) (Supplementary Figure S7) (25, 46). The coefficients of −0.0564 (*P* = 0.8288) and −4.1686(*P* = 0.2784) estimated by REML method were not statistically significant. There was no publication bias (*P-*liner = 0.2403, *P-*rank = 0.5730). The sensitivity analysis was shown in (Supplementary Figure S8). The maximum predictive effect for SA was achieved at 2.5 servings/day of dairy consumption, with the RR and 95% CI of 0.00 (0.00, 150.40).

### Hypospadias

In addition, we pooled three results from two studies and found that maternal rarely consumption of dairy products during pregnancy has a potential risk of hypospadias in offspring (RR = 1.27, 95% CI = 1.00, 1.60). (Supplementary Figure S9) (35, 42).

## Discussion

The present systematic review and meta-analyses comprehensively summarized the current updated evidence on the associations between maternal consumption of milk or dairy during pregnancy and birth outcomes. There were more than 20 birth outcomes involved in the present systematic review, and eight outcomes that could be meta-analyzed, covering birth anthropometrics, congenital malformation, and other adverse birth events. Overall, the present findings suggested that maternal dairy consumption during pregnancy had a growth-promoting effect on offspring, including a marginal significant effect on birth anthropometrics, a significant protective effect on SGA, and a risk effect on LGA; in addition, low consumption of organic dairy products might have an adverse effect on hypospadias in offspring. Our findings still need to be constantly updated with respect to improved sample size because neither of them is particularly strong.

To the best of our knowledge, this is the first study to quantitatively evaluate such associations using dose-response meta-analysis methods. Although the outcomes of our study partially overlap with those of previous systematic reviews, the focuses of these reviews are not identical (16, 17). Furthermore, compared with the previous systematic reviews, we not only conducted meta-analyses, but also discussed the dose-response relationships between dairy consumption and birth outcomes (16, 17). Particularly, in addition to dose-response meta-analysis for categorical variables, the method of the dose-response meta-analysis of differences in means was also applied (22). Moreover, the present study used the one-stage approach that no longer excluded studies with fewer than three exposure groups, and thus more relevant studies were included for aggregating data (23).

### Maternal Dairy Consumption and Birth Anthropometrics

BW-related studies accounted for a large proportion of all included birth outcomes. The present findings for BW are suggestive of growth-promoting effects of maternal dairy intake, which are largely consistent with those of previous systematic reviews (15–17). In addition, our findings further showed that BW increased but gradually slowed down with the increasing maternal dairy consumption, indicating that the BW-promoting effect of maternal dairy consumption was not linear. A significant dose-response relationship was also found with BL. Interestingly, this relationship curve increased first and then decreased, with BL peaking when dairy consumption reached one serving of dairy products per day. It suggests that there appears to be an optimal dose of dairy consumption during pregnancy for BL promotion, which may be a novel finding in contrast to prior studies (15–17). According to the global review of food-based dietary guidelines by Cámara et al., (63) the recommended intake of dairy products ranged from about two to four servings per day. At this dose range, our pooled curve shows a slow increase in BW, but the effect on BL cannot be estimated because the dose is outside the predicted limit of the BL curve. Nevertheless, our findings support the possible role of dairy consumption in promoting birth anthropometrics, and further determination of the optimal dose of maternal dairy consumption for offspring's growth and development is needed in the future.

### Maternal Dairy Consumption and Adverse Birth Outcomes

As expected, we found a protective effect on SGA and a risk effect on LGA with increasing dairy consumption. However, our findings on LBW are inconsistent with the previous non-dose-response meta-analysis, which found a reduction in LBW risk associated with increased dairy consumption (17). Although our findings indicated that the dose-response relationship between maternal dairy consumption and LBW was not significant, the pooled curve showed a trend that dairy consumption gradually became a risk factor from a protective factor. It again supports our hypothesis that there is an optimal intake of dairy products for growth promotion.

The present results indicated that dairy consumption in pregnant women was not associated with PB. This finding updates the previous systematic reviews, one of which was prevented for drawing any conclusions due to lack of studies (16); the other was a non-dose-response meta-analysis of a small sample (17). To our knowledge, two studies have found a link between dairy intake and PB under certain conditions, suggesting that the association between dairy intake and PB may be related to the type of dairy products, classification of PB, and weight of the pregnant woman (49, 52).

Additionally, the potential protective effect of maternal dairy products consumed against SA was suggested by our synthesized data; however, this effect was based on a limited sample analysis and needed to be further determined. Surprisingly, although the primary studies were not statistically significant, our pooled results indicated a significant adverse effect of low maternal dairy intake on hypospadias in male offspring (35, 42). The exposures in both primary studies were organic dairy, so this finding suggests that the effects of organic dairy products may need to be treated differently from those of conventional dairy products.

### Mechanisms

Mechanisms underlying the relationship of milk and dairy consumption with birth anthropometrics may be biologically plausible based on current evidence. A growing body of evidence supports the positive effect of milk proteins on growth and developments (64, 65). Besides, insulin-like growth factor-1 (IGF-1), which is associated with milk intake, has been proposed as a candidate for regulating the fetal growth (66, 67). Furthermore, nutrient-sensitive kinase mechanistic target of rapamycin complex 1 pathway (mTORC1) is another promising growth regulator (68, 69). As evidence suggested, milk provides all signals for mTORC1-activation, and the activated mTORC1 pathway can facilitate anabolism and growth (70). Despite these potential biological clues, more clear evidence is still needed to underlie the association between maternal dairy consumption and fetal growth.

The mechanism of low organic dairy consumption as a risk factor for hypospadias in offspring is ambiguous. Based on current evidence, there are following hypotheses. The first hypothesis is related to the nutritionally relevant composition differences between organically and conventionally produced foods (71), just as reported, organic dairy products may provide additional benefits for health (72). Another possible mechanism is related to the lower level of pesticide residues in organic dairy products (73). As evidence shows, pesticides can increase the risk of hypospadias (74, 75), and thus it is speculated that consumption of organic dairy products may reduce the risk of hypospadias.

### Limitations

This large systematic review provides important insights into the effects of maternal dairy consumption on birth outcomes; nonetheless, several limitations should be considered. First, there are three limitations regarding the exposure: the examined exposure in the present systematic review was total dairy product rather than individual dairy products; dairy consumption was all during pregnancy but not the same “trimester;” the dose units for dairy consumption were unified according to the average size of several portion sizes. These issues are all attributed to the small number of homogeneous primary studies. Second, the limited number of studies and different data types prevented drawing any conclusions about other birth outcomes, such as head circumference, Apgar scores, and some congenital diseases. Third, in order to improve the sample size, we meta-analyzed the data regardless of the study design and study quality, which may increase the risk of bias for this systematic review. Therefore, our findings should be interpreted with caution and more well-designed studies are needed to update the conclusions of the present systematic review.

## Conclusion

In conclusion, the present systematic review is the first to evaluate the dose-response associations between maternal milk or dairy consumption and birth outcomes. The present findings, including the non-linear effect of maternal dairy consumption on birth anthropometrics, the preventive effect on SGA, and the risk effect on LGA, suggest a growth-promoting effect of dairy consumption during pregnancy, and the optimal dosage of dairy consumption needs to be further determined. Despite a small number of studies, the findings that low consumption of organic dairy products may increase the risk of hypospadias in male offspring should be taken seriously, and the conclusion still needs to be further updated. Further well-designed studies are warranted to clarify the specific roles of individual dairy products.

## Data Availability Statement

The raw data supporting the conclusions of this article will be made available by the authors, without undue reservation.

## Author Contributions

YZ: conceptualization, supervision, and funding acquisition. DH: conceptualization, resources, investigation, validation, methodology, software, formal analysis, writing—original draft, and writing—review and editing. QW: conceptualization, methodology, software, investigation, data curation, writing—review and editing, and project administration. XX: investigation, writing—review and editing, and project administration. CJ: resources, methodology, and formal analysis. YX: methodology, software, and validation. ZZ: methodology, software, and formal analysis. HD: investigation and formal analysis. HL: validation and visualization. SG: investigation and writing—original draft. QC: resources and data curation. All authors contributed to the article and approved the submitted version.

## Funding

This work was supported by the National Key R&D Program of China [grant number 2017YFC0907403, 2017].

## Conflict of Interest

The authors declare that the research was conducted in the absence of any commercial or financial relationships that could be construed as a potential conflict of interest.

## Publisher's Note

All claims expressed in this article are solely those of the authors and do not necessarily represent those of their affiliated organizations, or those of the publisher, the editors and the reviewers. Any product that may be evaluated in this article, or claim that may be made by its manufacturer, is not guaranteed or endorsed by the publisher.

## Abbreviations

PB: pre-mature birth
SA: spontaneous abortion
ML: maximum likelihood
BW: birth weight
BL: birth length
LBW: low birth weight
SGA: small for gestational age
LGA: large for gestational age
RR: Relative risk
CI: Confidence interval.
