# Supplementary material for: Maternal Consumption of Milk or Dairy Products During Pregnancy and Birth Outcomes: A Systematic Review and Dose-Response Meta-Analysis
Source: Front Nutr. 2022 Jun 9;9:900529. doi: 10.3389/fnut.2022.900529 (PMC9261982; doi:10.3389/fnut.2022.900529)
Supplement: Supplementary file 1 [file Data_Sheet_1.docx]

Supplementary Material

# Supplementary Material 1. The full search strategy

**Search strategy for PubMed (2019-05-23)**

1. "Dairy Products"[MeSH Terms] (90195)
2. Dairy Products (96856)
3. Dairy Product (97829)
4. Dairy (70768)
5. “Butter”[MeSH Terms] (1431)
6. Butter (5182)
7. “Cultured Milk Products”[MeSH Terms] (8862)
8. Cultured Milk Products (9157)
9. Cultured Milk Product (9249)
10. Milk Products (17466)
11. Milk Product (5567)
12. “Cheese”[MeSH Terms] (6157)
13. Cheese (11275)
14. “Yogurt”[MeSH Terms] (1944)
15. Yogurt (3425)
16. “Ice Cream”[MeSH Terms] (629)
17. Ice Cream (1414)
18. "Milk"[MeSH Terms] (77919)
19. Milk (135600)
20. **#1 OR #2 OR #3 OR #4 OR #5 OR #6 OR #7 OR #8 OR #9 OR #10 OR #11 OR #12 OR #13 OR #14 OR #15 OR #16 OR #17 OR #18 OR #19 (184820)**
21. "Eating"[MeSH Terms] (69911)
22. Eating (138865)
23. "Drinking"[MeSH Terms] (13872)
24. Drinking (160127)
25. Intake (265329)
26. Consumption (1080555)
27. **#21 OR #22 OR #23 OR #24 OR #25 OR #26 (1479610)**
28. “Birth Weight”[MeSH Terms] (39738)
29. Birth weight (103719)
30. “Infant, Low Birth Weight”[MeSH Terms] (32575)
31. Infant, Low Birth Weight (44426)
32. Low Birth Weight (53703)
33. “Infant, Small for Gestational Age”[MeSH Terms] (6840)
34. Infant, Small for Gestational Age (12274)
35. Small for Gestational Age (17395)
36. SGA (7524)
37. “Infant, Very Low Birth Weight”[MeSH Terms] (9528)
38. Infant, Very Low Birth Weight (39552)
39. “Infant, Extremely Low Birth Weight”[MeSH Terms] (1721)
40. Infant, Extremely Low Birth Weight (4208)
41. Infant, Large for Gestational Age (4515)
42. Large for Gestational Age (8179)
43. LGA (2072)
44. “Fetal Growth Retardation”[MeSH Terms] (15582)
45. Fetal Growth Retardation (19066)
46. Intrauterine Growth Retardation (20482)
47. Growth Retardation, Intrauterine (20482)
48. Intrauterine Growth Restriction (21956)
49. Fetal Growth Restriction (23569)
50. FGR (1658)
51. IUGR (20424)
52. “Premature Birth”[MeSH Terms] (12239)
53. Premature Birth (49225)
54. “Infant, Premature”[MeSH Terms] (52755)
55. Infant, Premature (87766)
56. Birth, Premature (49225)
57. Births, Premature (50489)
58. Premature Births (50489)
59. Preterm Birth (60956)
60. Birth, Preterm (60956)
61. Births, Preterm (51899)
62. Preterm Births (51899)
63. “Congenital, Hereditary, and Neonatal Diseases and Abnormalities”[MeSH Terms] (1168195)
64. Congenital, Hereditary, and Neonatal Diseases and Abnormalities (1168203)
65. Congenital Disorder (1175348)
66. Abnormality, Congenital (604366)
67. Congenital Abnormality (604366)
68. Deformities (959262)
69. Deformity (636060)
70. Congenital Defects (945432)
71. Congenital Defect (608115)
72. Defect, Congenital (608115)
73. Defects, Congenital (611152)
74. Abnormalities, Congenital (601910)
75. Birth Defects (946827)
76. Birth Defect (604706)
77. Defect, Birth (604706)
78. Defects, Birth (613591)
79. “Abortion, Spontaneous”[MeSH Terms] (34111)
80. Abortion, Spontaneous(38282)
81. Spontaneous abortion (38282)
82. “Abortion, Threatened”[MeSH Terms] (2249)
83. Abortion, Threatened (2868)
84. Miscarriage (43520)
85. “Apgar Score”[MeSH Terms] (7497)
86. Apgar Score (11630)
87. Score, Apgar (11630)
88. “Fetal Development”[MeSH Terms] (88669)
89. Fetal Development (525503)
90. Fetal Growth (193656)
91. “Anthropometry”[MeSH Terms] (490312)
92. Anthropometry (494104)
93. “Body Size”[MeSH Terms] (466375)
94. Body Size (529201)
95. Ponderal Index (1169)
96. Placental weight (16167)
97. Birth Length (17869)
98. Crown-Heel Length (251)
99. Head Circumference (7503)
100. Biparietal Diameter (1727)
101. “Skinfold Thickness”[MeSH Terms] (5992)
102. Skinfold Thickness (8061)
103. Triceps Skinfold (2854)
104. Mid-upper-arm Circumference (1109)
105. Fetal Abdominal Circumference (1449)
106. Fetal Femur Length (1194)
107. Birth Outcome (55368)
108. Obstetrical Outcome (63042)
109. **#28 OR #29 OR #30 OR #31 OR #32 OR #32 OR #33 OR #34 OR #35 OR #36 OR #37 OR #38 OR #39 OR #40 OR #41 OR #42 OR #43 OR #44 OR #45 OR #46 OR #47 OR #48 OR #49 OR #50 OR #51 OR #52 OR #53 OR #54 OR #55 OR #56 OR #57 OR #58 OR #59 OR #60 OR #61 OR #62 OR #63 OR #64 OR #65 OR #66 OR #67 OR #68 OR #69 OR #70 OR #71 OR #72 OR #73 OR #74 OR #75 OR #76 OR #77 OR #78 OR #79 OR #80 OR #81 OR #82 OR #83 OR #84 OR #85 OR #86 OR #87 OR #88 OR #89 OR #90 OR #91 OR #92 OR #93 OR #94 OR #95 OR #96 OR #97 OR #98 OR #99 OR #100 OR #101 OR #102 OR #103 OR #104 OR #105 OR #106 OR #107 OR #108 (2640843)**
110. “Pregnancy”[MeSH Terms] (861586)
111. Pregnancy (939248)
112. “Pregnant Women” [MeSH Terms] (7444)
113. Pregnant Women (107264)
114. Pregnant Woman (114564)
115. Women, Pregnant (107264)
116. Woman, Pregnant (114564)
117. Gestation (965961)
118. **#110 OR #111 OR #112 OR #113 OR #114 OR #115 OR #116 OR #117 (977620)**
119. **#20 AND #27 AND #109 AND #118 (2088)**

**Search strategy for Web of Science (2019-05-23)**

1. TS=(Dairy Products) (110024)
2. TS=(Dairy Product) (110024)
3. TS=(Dairy) (233438)
4. TS=(Butter) (15637)
5. TS=(Cultured Milk Products) (14862)
6. TS=(Cultured Milk Product) (14862)
7. TS=(Milk Products) (111316)
8. TS=(Milk Product) (111316)
9. TS=(Cheese) (35446)
10. TS=(Yogurt) (10926)
11. TS=(Ice Cream) (4364)
12. TS=(Milk) (299811)
13. **#1 OR #2 OR #3 OR #4 OR #5 OR #6 OR #7 OR #8 OR #9 OR #10 OR #11 OR #12 (386263)**
14. TS=(Eating) (242950)
15. TS=(Drinking) (299711)
16. TS=(Intake) (486489)
17. TS=(Consumption) (740346)
18. **#14 OR #15 OR #16 OR #17 (1472848)**
19. TS=(Birth Weight) (158795)
20. TS=(Infant, Low Birth Weight) (64967)
21. TS=(Low Birth Weight) (88497)
22. TS=(Infant, Small for Gestational Age) (15378)
23. TS=( Small for Gestational Age) (21983)
24. TS=(SGA) (9780)
25. TS=(Infant, Very Low Birth Weight) (17949)
26. TS=(Infant, Extremely Low Birth Weight) (6544)
27. TS=(Infant, Large for Gestational Age) (7162)
28. TS=(Large for Gestational Age) (11780)
29. TS=(LGA) (2964)
30. TS=(Fetal Growth Retardation) (24957)
31. TS=(Intrauterine Growth Retardation) (16088)
32. TS=(Growth Retardation, Intrauterine) (16088)
33. TS=(Intrauterine Growth Restriction) (12355)
34. TS=(Fetal Growth Restriction) (16735)
35. TS=(FGR) (2510)
36. TS=(IUGR) (7606)
37. TS=(Premature Birth) (69770)
38. TS=(Infant, Premature) (107090)
39. TS=(Birth, Premature) (69770)
40. TS=(Births, Premature) (69770)
41. TS=(Premature Births) (69770)
42. TS=(Preterm Birth) (65315)
43. TS=(Birth, Preterm) (65315)
44. TS=(Births, Preterm) (65315)
45. TS=(Preterm Births) (65315)
46. TS=(Congenital, Hereditary, and Neonatal Diseases and Abnormalities) (1139)
47. TS=(Congenital Disorder) (228949)
48. TS=(Abnormality, Congenital) (180100)
49. TS=(Congenital Abnormality) (180100)
50. TS=(Deformities) (98025)
51. TS=(Deformity) (98025)
52. TS=(Congenital Defects) (152913)
53. TS=(Congenital Defect) (152913)
54. TS=(Defect, Congenital) (152913)
55. TS=(Defects, Congenital) (152913)
56. TS=(Abnormalities, Congenital) (180100)
57. TS=(Birth Defects) (39651)
58. TS=(Birth Defect) (39651)
59. TS=(Defect, Birth) (39651)
60. TS=(Defects, Birth) (39651)
61. TS=(Abortion, Spontaneous) (41386)
62. TS=(Spontaneous abortion) (41386)
63. TS=(Abortion, Threatened) (4099)
64. TS=(Miscarriage) (20864)
65. TS=(Apgar Score) (17710)
66. TS=(Score, Apgar) (17710)
67. TS=(Fetal Development) (240196)
68. TS=(Fetal Growth) (112477)
69. TS=(Anthropometry) (55049)
70. TS=(Body Size) (335588)
71. TS=(Ponderal Index) (1795)
72. TS=(Placental weight) (15545)
73. TS=(Birth Length) (27198)
74. TS=(Crown-Heel Length) (350)
75. TS=(Head Circumference) (10811)
76. TS=(Biparietal Diameter) (2201)
77. TS=(Skinfold Thickness) (10308)
78. TS=(Triceps Skinfold) (3912)
79. TS=(Mid-upper-arm Circumference) (1414)
80. TS=(Fetal Abdominal Circumference) (1838)
81. TS=(Fetal Femur Length) (1582)
82. TS=(Birth outcome) (105154)
83. TS=(Obstetrical outcome) (7453)
84. **#19 OR #20 OR #21 OR #22 OR #23 OR #24 OR #25 OR #26 OR #27 OR #28 OR #29 OR #30 OR #31 OR #32 OR #33 OR #34 OR #35 OR #36 OR #37 OR #38 OR #39 OR #40 OR #41 OR #42 OR #43 OR #44 OR #45 OR #46 OR #47 OR #48 OR #49 OR #50 OR #51 OR #52 OR #53 OR #54 OR #55 OR #56 OR #57 OR #58 OR #59 OR #60 OR #61 OR #62 OR #63 OR #64 OR #65 OR #66 OR #67 OR #68 OR #69 OR #70 OR #71 OR #72 OR #73 OR #74 OR #75 OR #76 OR #77 OR #78 OR #79 OR #80 OR #81 OR #82 OR #83 (1435558)**
85. TS=(Pregnancy) (1078513)
86. TS=(Pregnant Women) (150842)
87. TS=(Pregnant Woman) (150842)
88. TS=(Women, Pregnant) (150842)
89. TS=(Woman, Pregnant) (150842)
90. TS=(Gestation) (208327)
91. **#85 OR #86 OR #87 OR #88 OR #89 OR #90 (1138412)**
92. **#13 AND #18 AND #84 AND #91 (2754)**

**Search strategy for Cochrane Library (2019-05-23)**

1. MeSH descriptor: [Dairy Products] explode all trees (3442)

2. MeSH descriptor: [Butter] explode all trees (92)

3. MeSH descriptor: [Cultured Milk Products] explode all trees (497)

4. MeSH descriptor: [Cheese] explode all trees (79)

5. MeSH descriptor: [Yogurt] explode all trees (327)

6. MeSH descriptor: [Ice Cream] explode all trees (24)

7. MeSH descriptor: [Milk] explode all trees (2592)

8. (Dairy products OR Dairy product OR Dairy OR Butter OR Cultured Milk Products OR Cultured Milk Product OR Milk Products OR Milk Product OR Cheese OR Yogurt OR Ice Cream OR Milk):ti,ab,kw (12212)

**9. #1 OR #2 OR #3 OR #4 OR #5 OR #6 OR #7 OR #8 (12423)**

10. MeSH descriptor: [Eating] explode all trees (3351)

11. MeSH descriptor: [Drinking] explode all trees (546)

12. Eating OR Drinking OR Intake OR Consumption:ti,ab,kw (94534)

**13. #10 OR #11 OR #12 (94813)**

14. MeSH descriptor: [Birth Weight] explode all trees (1572)

15. MeSH descriptor: [Infant, Low Birth Weight] explode all trees (2077)

16. MeSH descriptor: [Infant, Small for Gestational Age] explode all trees (258)

17. MeSH descriptor: [Infant, Very Low Birth Weight] explode all trees (898)

18. MeSH descriptor: [Infant, Extremely Low Birth Weight] explode all trees (109)

19. MeSH descriptor: [Fetal Growth Retardation] explode all trees (353)

20. MeSH descriptor: [Premature Birth] explode all trees (1117)

21. MeSH descriptor: [Infant, Premature] explode all trees (3490)

22. MeSH descriptor: [Congenital, Hereditary, and Neonatal Diseases and Abnormalities] explode all trees (19483)

23. MeSH descriptor: [Abortion, Spontaneous] explode all trees (800)

24. MeSH descriptor: [Abortion, Threatened] explode all trees (52)

25. MeSH descriptor: [Apgar Score] explode all trees(666)

26. MeSH descriptor: [Fetal Development] explode all trees (2573)

27. MeSH descriptor: [Anthropometry] explode all trees (21890)

28. MeSH descriptor: [Body Size] explode all trees (25583)

29. MeSH descriptor: [Skinfold Thickness] explode all trees (320)

30. (Birth weight OR Infant, Low Birth Weight OR Low Birth Weight OR Infant, Small for Gestational Age OR Small for Gestational Age OR SGA OR Infant, Very Low Birth Weight OR Infant, Extremely Low Birth Weight OR Infant, Large for Gestational Age OR Large for Gestational age OR LGA):ti,ab,kw (13059)

31. (Fetal Growth Retardation OR Intrauterine Growth Retardation OR Growth Retardation, Intrauterine OR Intrauterine Growth Restriction OR Fetal Growth Restriction OR FGR OR IUGR):ti,ab,kw (1304)

32. (Premature Birth OR Infant, Premature OR Birth, Premature OR Births, Premature OR Premature Births OR Preterm Birth OR Birth, Preterm OR Births, Preterm OR Preterm Births):ti,ab,kw (11289)

33. (Congenital, Hereditary, and Neonatal Diseases and Abnormalities OR Congenital Disorder OR Abnormality, Congenital OR Congenital Abnormality OR Deformities OR Deformity OR Congenital Defects OR Congenital Defect OR Defect, Congenital OR Defects, Congenital OR Abnormalities, Congenital OR Birth Defects OR Birth Defect OR Defect, Birth OR Defects, Birth):ti,ab,kw (7409)

34. (Abortion, Spontaneous OR Spontaneous abortion OR Abortion, Threatened OR Miscarriage):ti,ab,kw (2860)

35. (Apgar Score OR Score, Apgar OR Fetal Development OR Fetal Growth OR Anthropometry OR Body Size OR Ponderal Index OR Placental weight OR Birth Length OR Crown-Heel Length OR Head Circumference OR Biparietal Diameter OR Skinfold Thickness OR Triceps Skinfold OR Mid-upper-arm Circumference OR Fetal Abdominal Circumference OR Fetal Femur Length OR Birth outcome OR Obstetrical outcome):ti,ab,kw (31512)

36. **#14 OR #15 OR #16 OR #17 OR #18 OR #19 OR #20 OR #21 OR #22 OR #23 OR #24 OR #25 OR #26 OR #27 OR #28 OR #29 OR #30 OR #31 OR #32 OR #32 OR #33 OR #34 OR #35 (89547)**

37. MeSH descriptor: [Pregnancy] explode all trees (7269)

38. MeSH descriptor: [Pregnant Women] explode all trees (203)

1. (Pregnancy OR Pregnant Women OR Pregnant Woman OR Women, Pregnant OR Woman, Pregnant OR Gestation):ti,ab,kw (57097)
2. **#37 OR #38 OR #39 (57305)**
3. **#9 AND #13 AND #36 AND #40 (181)**

**Search strategy for Embase (2019-05-23)**

1. ‘dairy product’/exp (113866)
2. ‘dairy products’ (11095)
3. ‘dairy product’ (13618)
4. ‘dairy’ (83684)
5. ‘butter’/exp (3898)
6. ‘butter’ (7914)
7. ‘cultured milk products’/exp (5851)
8. ‘cultured milk products’ (21)
9. ‘cultured milk product’ (3)
10. ‘milk products’ (4285)
11. ‘milk product’ (584)
12. ‘cheese’/exp (10513)
13. ‘cheese’ (14738)
14. ‘yogurt’/exp (5073)
15. ‘yogurt’ (3489)
16. ‘ice cream’/exp (1424)
17. ‘ice cream’ (2012)
18. ‘milk’/exp (81376)
19. ‘milk’ (175895)
20. **#1 OR #2 OR #3 OR #4 OR #5 OR #6 OR #7 OR #8 OR #9 OR #10 OR #11 OR #12 OR #13 OR #14 OR #15 OR #16 OR #17 OR #18 OR #19 (241160)**
21. ‘eating’/exp (34742)
22. ‘eating’ (124230)
23. ‘drinking’/exp (25724)
24. ‘drinking’ (182785)
25. ‘intake’ (540991)
26. ‘consumption’ (508218)
27. **#21 OR #22 OR #23 OR #24 OR #25 OR #26 (1133956)**
28. ‘birth weight’/exp (116142)
29. ‘birth weight’ (124033)
30. ‘infant, low birth weight’/exp (57923)
31. ‘infant, low birth weight’ (226)
32. ‘low birth weight’ (54641)
33. ‘infant, small for gestational age’/exp (13950)
34. ‘infant, small for gestational age’ (85)
35. ‘small for gestational age’ (12763)
36. ‘sga’ (12447)
37. ‘infant, very low birth weight’/exp (13948)
38. ‘infant, very low birth weight’ (78)
39. ‘infant, extremely low birth weight’/exp (3011)
40. ‘infant, extremely low birth weight’ (11)
41. ‘infant, large for gestational age’ (6)
42. ‘large for gestational age’ (3755)
43. ‘lga’ (3331)
44. ‘fetal growth retardation’/exp (40666)
45. ‘fetal growth retardation’ (2096)
46. ‘intrauterine growth retardation’ (28528)
47. ‘growth retardation, intrauterine’ (33)
48. ‘intrauterine growth restriction’ (8697)
49. ‘fetal growth restriction’ (6119)
50. ‘fgr’ (2735)
51. ‘iugr’ (9157)
52. ‘premature birth’/exp (108279)
53. ‘premature birth’ (5428)
54. ‘infant, premature’/exp (108279)
55. ‘infant, premature’ (1361)
56. ‘birth, premature’ (116)
57. ‘births, premature’ (30)
58. ‘premature births’ (1474)
59. ‘preterm birth’ (22814)
60. ‘birth, preterm’ (296)
61. ‘births, preterm’ (74)
62. ‘preterm births’ (3571)
63. ‘congenital, hereditary, and neonatal diseases and abnormalities’/exp (1677678)
64. ‘congenital, hereditary, and neonatal diseases and abnormalities’ (23)
65. ‘congenital disorder’ (294773)
66. ‘abnormality, congenital’ (26)
67. ‘congenital abnormality’ (2908)
68. ‘deformities’ (38954)
69. ‘deformity’ (74441)
70. ‘congenital defects’ (5448)
71. ‘congenital defect’ (2170)
72. ‘defect, congenital’ (117)
73. ‘defects, congenital’ (4166)
74. ‘abnormalities, congenital’ (203)
75. ‘birth defects’ (23162)
76. ‘birth defect’ (3988)
77. ‘defect, birth’ (15)
78. ‘defects, birth’ (28)
79. ‘abortion, spontaneous’/exp (40886)
80. ‘abortion, spontaneous’ (171)
81. ‘spontaneous abortion’ (43632)
82. ‘abortion, threatened’/exp (3269)
83. ‘abortion, threatened’ (68)
84. ‘miscarriage’ (17410)
85. ‘apgar score’/exp (22414)
86. ‘apgar score’ (24541)
87. ‘score, apgar’ (17)
88. ‘fetal development’/exp (27632)
89. ‘fetal development’ (11877)
90. ‘fetal growth’ (19950)
91. ‘anthropometry’/exp (80721)
92. ‘anthropometry’ (56847)
93. ‘body size’/exp (25254)
94. ‘body size’ (34497)
95. ‘ponderal index’ (1414)
96. ‘placental weight’ (2760)
97. ‘birth length’ (1841)
98. ‘crown-heel length’ (331)
99. ‘head circumference’ (14445)
100. ‘biparietal diameter’ (2102)
101. ‘skinfold thickness’/exp (12781)
102. ‘skinfold thickness’ (14397)
103. ‘triceps skinfold’ (2206)
104. ‘mid-upper-arm circumference’ (1399)
105. ‘fetal abdominal circumference’ (297)
106. ‘fetal femur length’ (126)
107. ‘birth outcome’ (1269)
108. ‘obstetrical outcome’ (376)
109. **#28 OR #29 OR #30 OR #31 OR #32 OR #32 OR #33 OR #34 OR #35 OR #36 OR #37 OR #38 OR #39 OR #40 OR #41 OR #42 OR #43 OR #44 OR #45 OR #46 OR #47 OR #48 OR #49 OR #50 OR #51 OR #52 OR #53 OR #54 OR #55 OR #56 OR #57 OR #58 OR #59 OR #60 OR #61 OR #62 OR #63 OR #64 OR #65 OR #66 OR #67 OR #68 OR #69 OR #70 OR #71 OR #72 OR #73 OR #74 OR #75 OR #76 OR #77 OR #78 OR #79 OR #80 OR #81 OR #82 OR #83 OR #84 OR #85 OR #86 OR #87 OR #88 OR #89 OR #90 OR #91 OR #92 OR #93 OR #94 OR #95 OR #96 OR #97 OR #98 OR #99 OR #100 OR #101 OR #102 OR #103 OR #104 OR #105 OR #106 OR #107 OR #108 (2075111)**
110. ‘pregnancy’/exp (760756)
111. ‘pregnancy’ (951837)
112. ‘pregnant women’/exp (76303)
113. ‘pregnant women’ (120077)
114. ‘pregnant woman’ (83970)
115. ‘women, pregnant’ (797)
116. ‘woman, pregnant’ (102)
117. ‘gestation’ (163448)
118. **#110 OR #111 OR #112 OR #113 OR #114 OR #115 OR #116 OR #117 (1029699)**
119. **#20 AND #27 AND #109 AND #118 (1229)**

**Supplementary search strategy for PubMed (2021-03-30)**

1. "Dairy Products"[MeSH Terms] (97404)
2. Dairy Products (105774)
3. Dairy Product (106978)
4. Dairy (83749)
5. “Butter”[MeSH Terms] (1478)
6. Butter (6694)
7. “Cultured Milk Products”[MeSH Terms] (9865)
8. Cultured Milk Products (10189)
9. Cultured Milk Product (10291)
10. Milk Products (46225)
11. Milk Product (46225)
12. “Cheese”[MeSH Terms] (6726)
13. Cheese (13205)
14. “Yogurt”[MeSH Terms] (2222)
15. Yogurt (5061)
16. “Ice Cream”[MeSH Terms] (668)
17. Ice Cream (1582)
18. "Milk"[MeSH Terms] (84168)
19. Milk (150006)
20. **#1 OR #2 OR #3 OR #4 OR #5 OR #6 OR #7 OR #8 OR #9 OR #10 OR #11 OR #12 OR #13 OR #14 OR #15 OR #16 OR #17 OR #18 OR #19 (209106)**
21. "Eating"[MeSH Terms] (74027)
22. Eating (154316)
23. "Drinking"[MeSH Terms] (14354)
24. Drinking (196124)
25. Intake (306266)
26. Consumption (1189052)
27. **#21 OR #22 OR #23 OR #24 OR #25 OR #26 (1649480)**
28. “Birth Weight”[MeSH Terms] (42230)
29. Birth weight (113302)
30. “Infant, Low Birth Weight”[MeSH Terms] (35294)
31. Infant, Low Birth Weight (48585)
32. Low Birth Weight (59286)
33. “Infant, Small for Gestational Age”[MeSH Terms] (7663)
34. Infant, Small for Gestational Age (13841)
35. Small for Gestational Age (19632)
36. SGA (9008)
37. “Infant, Very Low Birth Weight”[MeSH Terms] (10480)
38. Infant, Very Low Birth Weight (43114)
39. “Infant, Extremely Low Birth Weight”[MeSH Terms] (1963)
40. Infant, Extremely Low Birth Weight (4766)
41. Infant, Large for Gestational Age (6185)
42. Large for Gestational Age (10442)
43. LGA (2629)
44. “Fetal Growth Retardation”[MeSH Terms] (16692)
45. Fetal Growth Retardation (20289)
46. Intrauterine Growth Retardation (21991)
47. Growth Retardation, Intrauterine (21991)
48. Intrauterine Growth Restriction (23836)
49. Fetal Growth Restriction (25590)
50. FGR (2111)
51. IUGR (21830)
52. “Premature Birth”[MeSH Terms] (14992)
53. Premature Birth (55432)
54. “Infant, Premature”[MeSH Terms] (57521)
55. Infant, Premature (95597)
56. Birth, Premature (55432)
57. Births, Premature (56797)
58. Premature Births (56797)
59. Preterm Birth (70270)
60. Birth, Preterm (70270)
61. Births, Preterm (58718)
62. Preterm Births (58718)
63. “Congenital, Hereditary, and Neonatal Diseases and Abnormalities”[MeSH Terms] (1255730)
64. Congenital, Hereditary, and Neonatal Diseases and Abnormalities (1255748)
65. Congenital Disorder (1263877)
66. Abnormality, Congenital (640605)
67. Congenital Abnormality (640605)
68. Deformities (1124906)
69. Deformity (1124906)
70. Congenital Defects (1006616)
71. Congenital Defect (644965)
72. Defect, Congenital (644965)
73. Defects, Congenital (648519)
74. Abnormalities, Congenital (637887)
75. Birth Defects (1008729)
76. Birth Defect (641238)
77. Defect, Birth (641238)
78. Defects, Birth (52589)
79. “Abortion, Spontaneous”[MeSH Terms] (35783)
80. Abortion, Spontaneous (40380)
81. Spontaneous abortion (40380)
82. “Abortion, Threatened”[MeSH Terms] (2286)
83. Abortion, Threatened (2950)
84. Miscarriage (48818)
85. “Apgar Score”[MeSH Terms] (7838)
86. Apgar Score (12619)
87. Score, Apgar (12619)
88. “Fetal Development”[MeSH Terms] (94732)
89. Fetal Development (553479)
90. Fetal Growth (205313)
91. “Anthropometry”[MeSH Terms] (525302)
92. Anthropometry (529977)
93. “Body Size”[MeSH Terms] (505527)
94. Body Size (576548)
95. Ponderal Index (1308)
96. Placental weight (18468)
97. Birth Length (21294)
98. Crown-Heel Length (267)
99. Head Circumference (8817)
100. Biparietal Diameter (1961)
101. “Skinfold Thickness”[MeSH Terms] (6130)
102. Skinfold Thickness (8579)
103. Triceps Skinfold (3465)
104. Mid-upper-arm Circumference (1414)
105. Fetal Abdominal Circumference (1732)
106. Fetal Femur Length (1373)
107. Birth Outcome (92465)
108. Obstetrical Outcome (109257)
109. **#28 OR #29 OR #30 OR #31 OR #32 OR #32 OR #33 OR #34 OR #35 OR #36 OR #37 OR #38 OR #39 OR #40 OR #41 OR #42 OR #43 OR #44 OR #45 OR #46 OR #47 OR #48 OR #49 OR #50 OR #51 OR #52 OR #53 OR #54 OR #55 OR #56 OR #57 OR #58 OR #59 OR #60 OR #61 OR #62 OR #63 OR #64 OR #65 OR #66 OR #67 OR #68 OR #69 OR #70 OR #71 OR #72 OR #73 OR #74 OR #75 OR #76 OR #77 OR #78 OR #79 OR #80 OR #81 OR #82 OR #83 OR #84 OR #85 OR #86 OR #87 OR #88 OR #89 OR #90 OR #91 OR #92 OR #93 OR #94 OR #95 OR #96 OR #97 OR #98 OR #99 OR #100 OR #101 OR #102 OR #103 OR #104 OR #105 OR #106 OR #107 OR #108 (2957360)**
110. “Pregnancy”[MeSH Terms] (912737)
111. Pregnancy (1013961)
112. “Pregnant Women” [MeSH Terms] (9178)
113. Pregnant Women (124437)
114. Pregnant Woman (132414)
115. Women, Pregnant (124437)
116. Woman, Pregnant (132414)
117. Gestation (1077696)
118. **#110 OR #111 OR #112 OR #113 OR #114 OR #115 OR #116 OR #117 (1097987)**
119. **#20 AND #27 AND #109 AND #118 (2821)**
120. **(#20 AND #27 AND #109 AND #118) AND (("2019/05/23"[Date - Publication] : "3000"[Date - Publication])) (254)**

**Supplementary search strategy for Web of Science (2021-03-30)**

1. TS=(Dairy Products) (124390)
2. TS=(Dairy Product) (124390)
3. TS=(Dairy) (263947)
4. TS=(Butter) (17745)
5. TS=(Cultured Milk Products) (17053)
6. TS=(Cultured Milk Product) (17053)
7. TS=(Milk Products) (126555)
8. TS=(Milk Product) (126555)
9. TS=(Cheese) (39847)
10. TS=(Yogurt) (12636)
11. TS=(Ice Cream) (4988)
12. TS=(Milk) (337126)
13. **#1 OR #2 OR #3 OR #4 OR #5 OR #6 OR #7 OR #8 OR #9 OR #10 OR #11 OR #12 (432208)**
14. TS=(Eating) (276632)
15. TS=(Drinking) (338087)
16. TS=(Intake) (554246)
17. TS=(Consumption) (875598)
18. **#14 OR #15 OR #16 OR #17 (1698757)**
19. TS=(Birth Weight) (173700)
20. TS=(Infant, Low Birth Weight) (71514)
21. TS=(Low Birth Weight) (97922)
22. TS=(Infant, Small for Gestational Age) (17302)
23. TS=( Small for Gestational Age) (23920)
24. TS=(SGA) (11422)
25. TS=(Infant, Very Low Birth Weight) (19596)
26. TS=(Infant, Extremely Low Birth Weight) (7316)
27. TS=(Infant, Large for Gestational Age) (8340)
28. TS=(Large for Gestational Age) (13716)
29. TS=(LGA) (3545)
30. TS=(Fetal Growth Retardation) (26485)
31. TS=(Intrauterine Growth Retardation) (16829)
32. TS=(Growth Retardation, Intrauterine) (16829)
33. TS=(Intrauterine Growth Restriction) (13424)
34. TS=(Fetal Growth Restriction) (18888)
35. TS=(FGR) (3085)
36. TS=(IUGR) (8476)
37. TS=(Premature Birth) (78472)
38. TS=(Infant, Premature) (117815)
39. TS=(Birth, Premature) (78472)
40. TS=(Births, Premature) (78472)
41. TS=(Premature Births) (78472)
42. TS=(Preterm Birth) (74347)
43. TS=(Birth, Preterm) (74347)
44. TS=(Births, Preterm) (74347)
45. TS=(Preterm Births) (74347)
46. TS=(Congenital, Hereditary, and Neonatal Diseases and Abnormalities) (1190)
47. TS=(Congenital Disorder) (262180)
48. TS=(Abnormality, Congenital) (194108)
49. TS=(Congenital Abnormality) (194108)
50. TS=(Deformities) (109425)
51. TS=(Deformity) (109425)
52. TS=(Congenital Defects) (169042)
53. TS=(Congenital Defect) (169042)
54. TS=(Defect, Congenital) (169042)
55. TS=(Defects, Congenital) (169042)
56. TS=(Abnormalities, Congenital) (194108)
57. TS=(Birth Defects) (45528)
58. TS=(Birth Defect) (45528)
59. TS=(Defect, Birth) (45528)
60. TS=(Defects, Birth) (45528)
61. TS=(Abortion, Spontaneous) (44820)
62. TS=(Spontaneous abortion) (44820)
63. TS=(Abortion, Threatened) (4330)
64. TS=(Miscarriage) (24534)
65. TS=(Apgar Score) (19434)
66. TS=(Score, Apgar) (19434)
67. TS=(Fetal Development) (256176)
68. TS=(Fetal Growth) (119891)
69. TS=(Anthropometry) (59275)
70. TS=(Body Size) (380999)
71. TS=(Ponderal Index) (1970)
72. TS=(Placental weight) (16986)
73. TS=(Birth Length) (30712)
74. TS=(Crown-Heel Length) (369)
75. TS=(Head Circumference) (12114)
76. TS=(Biparietal Diameter) (2370)
77. TS=(Skinfold Thickness) (10726)
78. TS=(Triceps Skinfold) (4137)
79. TS=(Mid-upper-arm Circumference) (1734)
80. TS=(Fetal Abdominal Circumference) (2068)
81. TS=(Fetal Femur Length) (1721)
82. TS=(Birth outcome) (123360)
83. TS=(Obstetrical outcome) (8495)
84. **#19 OR #20 OR #21 OR #22 OR #23 OR #24 OR #25 OR #26 OR #27 OR #28 OR #29 OR #30 OR #31 OR #32 OR #33 OR #34 OR #35 OR #36 OR #37 OR #38 OR #39 OR #40 OR #41 OR #42 OR #43 OR #44 OR #45 OR #46 OR #47 OR #48 OR #49 OR #50 OR #51 OR #52 OR #53 OR #54 OR #55 OR #56 OR #57 OR #58 OR #59 OR #60 OR #61 OR #62 OR #63 OR #64 OR #65 OR #66 OR #67 OR #68 OR #69 OR #70 OR #71 OR #72 OR #73 OR #74 OR #75 OR #76 OR #77 OR #78 OR #79 OR #80 OR #81 OR #82 OR #83 (1590890)**
85. TS=(Pregnancy) (1165554)
86. TS=(Pregnant Women) (175634)
87. TS=(Pregnant Woman) (175634)
88. TS=(Women, Pregnant) (175634)
89. TS=(Woman, Pregnant) (175634)
90. TS=(Gestation) (241717)
91. **#85 OR #86 OR #87 OR #88 OR #89 OR #90 (1231550)**
92. **#13 AND #18 AND #84 AND #91 (3109)**

**93. #13 AND #18 AND #84 AND #91 AND PY=(2019-2021) (344)**

**Supplementary search strategy for Cochrane Library (2021-03-30)**

1. MeSH descriptor: [Dairy Products] explode all trees (3752)

2. MeSH descriptor: [Butter] explode all trees (92)

3. MeSH descriptor: [Cultured Milk Products] explode all trees (539)

4. MeSH descriptor: [Cheese] explode all trees (87)

5. MeSH descriptor: [Yogurt] explode all trees (355)

6. MeSH descriptor: [Ice Cream] explode all trees (24)

7. MeSH descriptor: [Milk] explode all trees (2845)

8. (Dairy products OR Dairy product OR Dairy OR Butter OR Cultured Milk Products OR Cultured Milk Product OR Milk Products OR Milk Product OR Cheese OR Yogurt OR Ice Cream OR Milk):ti,ab,kw (14567)

**9. #1 OR #2 OR #3 OR #4 OR #5 OR #6 OR #7 OR #8 (14842)**

10. MeSH descriptor: [Eating] explode all trees (3593)

11. MeSH descriptor: [Drinking] explode all trees (578)

12. Eating OR Drinking OR Intake OR Consumption:ti,ab,kw (112138)

**13. #10 OR #11 OR #12 (112449)**

14. MeSH descriptor: [Birth Weight] explode all trees (1751)

15. MeSH descriptor: [Infant, Low Birth Weight] explode all trees (2214)

16. MeSH descriptor: [Infant, Small for Gestational Age] explode all trees (284)

17. MeSH descriptor: [Infant, Very Low Birth Weight] explode all trees (970)

18. MeSH descriptor: [Infant, Extremely Low Birth Weight] explode all trees (119)

19. MeSH descriptor: [Fetal Growth Retardation] explode all trees (399)

20. MeSH descriptor: [Premature Birth] explode all trees (1511)

21. MeSH descriptor: [Infant, Premature] explode all trees (3820)

22. MeSH descriptor: [Congenital, Hereditary, and Neonatal Diseases and Abnormalities] explode all trees (22993)

23. MeSH descriptor: [Abortion, Spontaneous] explode all trees (907)

24. MeSH descriptor: [Abortion, Threatened] explode all trees (57)

25. MeSH descriptor: [Apgar Score] explode all trees(690)

26. MeSH descriptor: [Fetal Development] explode all trees (2785)

27. MeSH descriptor: [Anthropometry] explode all trees (23534)

28. MeSH descriptor: [Body Size] explode all trees (29300)

29. MeSH descriptor: [Skinfold Thickness] explode all trees (321)

30. (Birth weight OR Infant, Low Birth Weight OR Low Birth Weight OR Infant, Small for Gestational Age OR Small for Gestational Age OR SGA OR Infant, Very Low Birth Weight OR Infant, Extremely Low Birth Weight OR Infant, Large for Gestational Age OR Large for Gestational age OR LGA):ti,ab,kw (14723)

31. (Fetal Growth Retardation OR Intrauterine Growth Retardation OR Growth Retardation, Intrauterine OR Intrauterine Growth Restriction OR Fetal Growth Restriction OR FGR OR IUGR):ti,ab,kw (1497)

32. (Premature Birth OR Infant, Premature OR Birth, Premature OR Births, Premature OR Premature Births OR Preterm Birth OR Birth, Preterm OR Births, Preterm OR Preterm Births):ti,ab,kw (12887)

33. (Congenital, Hereditary, and Neonatal Diseases and Abnormalities OR Congenital Disorder OR Abnormality, Congenital OR Congenital Abnormality OR Deformities OR Deformity OR Congenital Defects OR Congenital Defect OR Defect, Congenital OR Defects, Congenital OR Abnormalities, Congenital OR Birth Defects OR Birth Defect OR Defect, Birth OR Defects, Birth):ti,ab,kw (7910)

34. (Abortion, Spontaneous OR Spontaneous abortion OR Abortion, Threatened OR Miscarriage):ti,ab,kw (3152)

35. (Apgar Score OR Score, Apgar OR Fetal Development OR Fetal Growth OR Anthropometry OR Body Size OR Ponderal Index OR Placental weight OR Birth Length OR Crown-Heel Length OR Head Circumference OR Biparietal Diameter OR Skinfold Thickness OR Triceps Skinfold OR Mid-upper-arm Circumference OR Fetal Abdominal Circumference OR Fetal Femur Length OR Birth outcome OR Obstetrical outcome):ti,ab,kw (37456)

36. **#14 OR #15 OR #16 OR #17 OR #18 OR #19 OR #20 OR #21 OR #22 OR #23 OR #24 OR #25 OR #26 OR #27 OR #28 OR #29 OR #30 OR #31 OR #32 OR #32 OR #33 OR #34 OR #35 (103519)**

37. MeSH descriptor: [Pregnancy] explode all trees (22290)

38. MeSH descriptor: [Pregnant Women] explode all trees (290)

39. (Pregnancy OR Pregnant Women OR Pregnant Woman OR Women, Pregnant OR Woman, Pregnant OR Gestation):ti,ab,kw (66169)

**40. #37 OR #38 OR #39 (66433)**

**41. #9 AND #13 AND #36 AND #40 (226)**

**42. #9 AND #13 AND #36 AND #40 with Cochrane Library publication date from May 2019 to Mar 2021 (53)**

**Supplementary search strategy for Embase (2021-03-30)**

1. ‘dairy product’/exp (116575)
2. ‘dairy products’ (12737)
3. ‘dairy product’ (15584)
4. ‘dairy’ (94236)
5. ‘butter’/exp (3426)
6. ‘butter’ (7631)
7. ‘cultured milk products’/exp (7173)
8. ‘cultured milk products’ (22)
9. ‘cultured milk product’ (2)
10. ‘milk products’ (4390)
11. ‘milk product’ (620)
12. ‘cheese’/exp (11261)
13. ‘cheese’ (15987)
14. ‘yogurt’/exp (5938)
15. ‘yogurt’ (6435)
16. ‘ice cream’/exp (1522)
17. ‘ice cream’ (2162)
18. ‘milk’/exp (80489)
19. ‘milk’ (184120)
20. **#1 OR #2 OR #3 OR #4 OR #5 OR #6 OR #7 OR #8 OR #9 OR #10 OR #11 OR #12 OR #13 OR #14 OR #15 OR #16 OR #17 OR #18 OR #19 (256494)**
21. ‘eating’/exp (37727)
22. ‘eating’ (141446)
23. ‘drinking’/exp (26153)
24. ‘drinking’ (199583)
25. ‘intake’ (592113)
26. ‘consumption’ (568692)
27. **#21 OR #22 OR #23 OR #24 OR #25 OR #26 (1251322)**
28. ‘birth weight’/exp (130210)
29. ‘birth weight’ (138017)
30. ‘infant, low birth weight’/exp (64678)
31. ‘infant, low birth weight’ (240)
32. ‘low birth weight’ (60262)
33. ‘infant, small for gestational age’/exp (16500)
34. ‘infant, small for gestational age’ (94)
35. ‘small for gestational age’ (15005)
36. ‘sga’ (14940)
37. ‘infant, very low birth weight’/exp (15570)
38. ‘infant, very low birth weight’ (85)
39. ‘infant, extremely low birth weight’/exp (3515)
40. ‘infant, extremely low birth weight’ (14)
41. ‘infant, large for gestational age’ (6)
42. ‘large for gestational age’ (4685)
43. ‘lga’ (4082)
44. ‘fetal growth retardation’/exp (46620)
45. ‘fetal growth retardation’ (2154)
46. ‘intrauterine growth retardation’ (32121)
47. ‘growth retardation, intrauterine’ (35)
48. ‘intrauterine growth restriction’ (9999)
49. ‘fetal growth restriction’ (7613)
50. ‘fgr’ (3430)
51. ‘iugr’ (10143)
52. ‘premature birth’/exp (116617)
53. ‘premature birth’ (5842)
54. ‘infant, premature’/exp (116617)
55. ‘infant, premature’ (1557)
56. ‘birth, premature’ (115)
57. ‘births, premature’ (28)
58. ‘premature births’ (1245)
59. ‘preterm birth’ (28063)
60. ‘birth, preterm’ (380)
61. ‘births, preterm’ (86)
62. ‘preterm births’ (4226)
63. ‘congenital, hereditary, and neonatal diseases and abnormalities’/exp (1755018)
64. ‘congenital, hereditary, and neonatal diseases and abnormalities’ (27)
65. ‘congenital disorder’ (307912)
66. ‘abnormality, congenital’ (23)
67. ‘congenital abnormality’ (2898)
68. ‘deformities’ (38458)
69. ‘deformity’ (75175)
70. ‘congenital defects’ (5031)
71. ‘congenital defect’ (1956)
72. ‘defect, congenital’ (139)
73. ‘defects, congenital’ (5331)
74. ‘abnormalities, congenital’ (216)
75. ‘birth defects’ (25983)
76. ‘birth defect’ (4707)
77. ‘defect, birth’ (16)
78. ‘defects, birth’ (28)
79. ‘abortion, spontaneous’/exp (45339)
80. ‘abortion, spontaneous’ (192)
81. ‘spontaneous abortion’ (48246)
82. ‘abortion, threatened’/exp (2801)
83. ‘abortion, threatened’ (64)
84. ‘miscarriage’ (20195)
85. ‘apgar score’/exp (25949)
86. ‘apgar score’ (28228)
87. ‘score, apgar’ (21)
88. ‘fetal development’/exp (28616)
89. ‘fetal development’ (12874)
90. ‘fetal growth’ (22625)
91. ‘anthropometry’/exp (87555)
92. ‘anthropometry’ (62752)
93. ‘body size’/exp (27396)
94. ‘body size’ (37653)
95. ‘ponderal index’ (1448)
96. ‘placental weight’ (2963)
97. ‘birth length’ (2097)
98. ‘crown-heel length’ (305)
99. ‘head circumference’ (16690)
100. ‘biparietal diameter’ (2201)
101. ‘skinfold thickness’/exp (14127)
102. ‘skinfold thickness’ (15791)
103. ‘triceps skinfold’ (2330)
104. ‘mid-upper-arm circumference’ (1731)
105. ‘fetal abdominal circumference’ (348)
106. ‘fetal femur length’ (135)
107. ‘birth outcome’ (1532)
108. ‘obstetrical outcome’ (424)
109. **#28 OR #29 OR #30 OR #31 OR #32 OR #32 OR #33 OR #34 OR #35 OR #36 OR #37 OR #38 OR #39 OR #40 OR #41 OR #42 OR #43 OR #44 OR #45 OR #46 OR #47 OR #48 OR #49 OR #50 OR #51 OR #52 OR #53 OR #54 OR #55 OR #56 OR #57 OR #58 OR #59 OR #60 OR #61 OR #62 OR #63 OR #64 OR #65 OR #66 OR #67 OR #68 OR #69 OR #70 OR #71 OR #72 OR #73 OR #74 OR #75 OR #76 OR #77 OR #78 OR #79 OR #80 OR #81 OR #82 OR #83 OR #84 OR #85 OR #86 OR #87 OR #88 OR #89 OR #90 OR #91 OR #92 OR #93 OR #94 OR #95 OR #96 OR #97 OR #98 OR #99 OR #100 OR #101 OR #102 OR #103 OR #104 OR #105 OR #106 OR #107 OR #108 (2184755)**
110. ‘pregnancy’/exp (793611)
111. ‘pregnancy’ (1013715)
112. ‘pregnant women’/exp (86779)
113. ‘pregnant women’ (133640)
114. ‘pregnant woman’ (94988)
115. ‘women, pregnant’ (854)
116. ‘woman, pregnant’ (112)
117. ‘gestation’ (173745)
118. **#110 OR #111 OR #112 OR #113 OR #114 OR #115 OR #116 OR #117 (1098051)**
119. **#20 AND #27 AND #109 AND #118 (1399)**
120. **#20 AND #27 AND #109 AND #118 AND [2019-2021]/py (219)**
