# Supplementary material for: Maternal Consumption of Milk or Dairy Products During Pregnancy and Birth Outcomes: A Systematic Review and Dose-Response Meta-Analysis
Source: Front Nutr. 2022 Jun 9;9:900529. doi: 10.3389/fnut.2022.900529 (PMC9261982; doi:10.3389/fnut.2022.900529)
Supplement: Supplementary file 2 [file Table_2.DOCX]

Supplementary Material

# Supplementary Material 2. Reasons for exclusions in full-text screening

| **No.** | **Papers excluded** | **Reasons for exclusion** |
| --- | --- | --- |
| 1 | Mardones-Santander, F., et al. (1988). "Effect of a milk-based food supplement on maternal nutritional status and fetal growth in underweight Chilean women." Am J Clin Nutr 47(3): 413-419. | The intervention examined was milk-based fortified product |
| 2 | Friel, J. K., et al. (1995). "Nutritional patterns of mothers of children with neural tube defects in Newfoundland." American journal of medical genetics 55(2): 195-199. | The dairy exposure was not during pregnancy |
| 3 | Wolff, C. B. and H. K. Wolff (1995). "Maternal eating patterns and birth weight of Mexican American infants." Nutr Health 10(2): 121-134. | The exposure examined was dietary pattern |
| 4 | Cosgrove, M. and D. P. Davies (1996). "Poor diet in pregnancy may be a proxy for some other hostile influence on fetal growth [8]." Br Med J 312(7044): 1478-1479. | Type of article was Letter |
| 5 | Godfrey, K., et al. (1996). "Maternal nutrition in early and late pregnancy in relation to placental and fetal growth." Bmj 312(7028): 410-414. | The exposure examined was dairy protein |
| 6 | Oldak, E. (1996). "[The evaluation of nonspecific immunoglobulin E in cord blood of newborns in relation to maternal cow's milk allergen intake during pregnancy]." Pediatr Pol 71(7): 591-594. | It was a Polish paper with an English abstract |
| 7 | Godfrey, K. M., et al. (1997). "Maternal birthweight and diet in pregnancy in relation to the infant's thinness at birth." Br J Obstet Gynaecol 104(6): 663-667. | The exposure examined was dairy protein |
| 8 | Shaw, G. M., et al. (1999). "Periconceptional nutrient intake and risk for neural tube defect-affected pregnancies." Epidemiology (Cambridge, Mass.) 10(6): 711-716. | The exposure was periconceptional nutrient intake |
| 9 | Ushiyama, Y., et al. (2002). "Nutrition during pregnancy may be associated with allergic diseases in infants." J Nutr Sci Vitaminol (Tokyo) 48(5): 345-351. | The outcome examined was allergic diseases in infants |
| 10 | Kramer, M. S. and R. Kakuma (2003). "Energy and protein intake in pregnancy." Cochrane Database Syst Rev(4): CD000032. | Type of article was Review |
| 11 | Takimoto, H., et al. (2003). "Nutritional status of pregnant and lactating women in Japan: A comparison with non-pregnant/non-lactating controls in the National Nutrition Survey." Journal of Obstetrics and Gynaecology Research 29(2): 96-103. | No birth outcomes involved |
| 12 | Gao, L. J., et al. (2004). "[A case-control study on the risk factors of central nervous system congenital malformations]." Zhonghua Liu Xing Bing Xue Za Zhi 25(9): 794-798. | The exposure examined was combined consumption of meat, eggs, beans and milk during pregnancy |
| 13 | Lagiou, P., et al. (2004). "Diet during pregnancy in relation to maternal weight gain and birth size." Eur J Clin Nutr 58(2): 231-237. | The exposure examined was energy and energy generating nutrients |
| 14 | Moore, V. M., et al. (2004). "Dietary composition of pregnant women is related to size of the baby at birth." J Nutr 134(7): 1820-1826. | The exposure was protein from dairy sources |
| 15 | Javaid, M. K., et al. (2005). "Maternal and seasonal predictors of change in calcaneal quantitative ultrasound during pregnancy." J Clin Endocrinol Metab 90(9): 5182-5187. | The outcome was maternal change in calcaneal quantitative ultrasound during pregnancy |
| 16 | Olsen, S. F., et al. (2005). "Association of milk consumption in pregnancy with increased foetal growth." Pediatr Res 58(5): 1120-1120. | Type of article was Meeting Abstract |
| 17 | (2006). "Milk consumption and birth weight." Indian pediatrics 43(7): 661. | Type of article was Note |
| 18 | Henriksen, T. (2006). "Nutrition and pregnancy outcome." Nutr Rev 64(5 Pt 2): S19-23; discussion S72-91. | Type of article was Review |
| 19 | Willers, S. M., et al. (2007). "Maternal food consumption during pregnancy and asthma, respiratory and atopic symptoms in 5-year-old children." Thorax 62(9): 773-779. | The outcomes were asthma, respiratory and allergic outcomes in 5-year-old children |
| 20 | Flieger, K. (2008). "Milk consumption during pregnancy increases birth weight." Aktuelle Ernahrungsmedizin 33(3): 93. | Type of article was Note |
| 21 | Flieger, K. (2008). "Milk consumption during pregnancy increases infant size at birth." Geburtshilfe und Frauenheilkunde 68(4): 342. | Type of article was Note |
| 22 | Kaiser, L., et al. (2008). "Position of the American Dietetic Association: nutrition and lifestyle for a healthy pregnancy outcome." J Am Diet Assoc 108(3): 553-561. | It was a Practice Guideline |
| 23 | Mardones, F., et al. (2008). "Effects of a dairy product fortified with multiple micronutrients and omega-3 fatty acids on birth weight and gestation duration in pregnant Chilean women." Public Health Nutr 11(1): 30-40. | The exposure examined was fortified powdered milk |
| 24 | Shapira, N. (2008). "Prenatal nutrition: a critical window of opportunity for mother and child." Womens Health (Lond) 4(6): 639-656. | Type of article was Review |
| 25 | Willers, S. M., et al. (2008). "Maternal food consumption during pregnancy and the longitudinal development of childhood asthma." Am J Respir Crit Care Med 178(2): 124-131. | The outcome examined was childhood asthma outcomes from 1 to 8 years of age |
| 26 | Melnik, B. (2009). "Milk consumption: Aggravating factor of acne and promoter of chronic diseases of Western societies: Perspektiven." JDDG - Journal of the German Society of Dermatology 7(4): 364-370. | Type of article was Review |
| 27 | Melnik, B. C. (2009). "Milk--the promoter of chronic Western diseases." Med Hypotheses 72(6): 631-639. | Type of article was Review |
| 28 | Pinto, E., et al. (2009). "Dietary intake and nutritional adequacy prior to conception and during pregnancy: A follow-up study in the north of Portugal." Public Health Nutr 12(7): 922-931. | No birth outcomes involved |
| 29 | Stuebe, A. M., et al. (2009). "Associations of diet and physical activity during pregnancy with risk for excessive gestational weight gain." Am J Obstet Gynecol 201(1): 58.e51-58. | The outcome was gestational weight gain |
| 30 | Pérez, M. I., et al. (2010). "Consumption of a dairy product enriched in docosahexaenoic acid (DHA, 22:6 N-3) during pregnancy and lactation does not affect somatometric parameters of children during the first year of LIF." Journal of Maternal-Fetal and Neonatal Medicine 23: 582. | Type of article was Conference Abstract;The exposure was dairy product enriched with fish oil |
| 31 | Pfefferle, P. I., et al. (2010). "Cord blood cytokines are modulated by maternal farming activities and consumption of farm dairy products during pregnancy: the PASTURE Study." J Allergy Clin Immunol 125(1): 108-115.e101-103. | The outcome was cord blood cytokines |
| 32 | Saito, K., et al. (2010). "Maternal meat and fat consumption during pregnancy and suspected atopic eczema in Japanese infants aged 3-4 months: the Osaka Maternal and Child Health Study." Pediatr Allergy Immunol 21(1 Pt 1): 38-46. | The outcome was suspected atopic eczema among Japanese infants aged 3-4 months. |
| 33 | Salmenhaara, M., et al. (2010). "Diet and weight gain characteristics of pregnant women with gestational diabetes." Eur J Clin Nutr 64(12): 1433-1440. | No birth outcomes involved |
| 34 | Yin, J., et al. (2010). "The association between maternal diet during pregnancy and bone mass of the children at age 16." Eur J Clin Nutr 64(2): 131-137. | The outcome was bone mass of the children at age 16 |
| 35 | Ford, J. H. (2011). "Preconception risk factors and SGA babies: Papilloma virus, omega 3 and fat soluble vitamin deficiencies." Early Hum Dev 87(12): 785-789. | The exposure examined was preconception food intake |
| 36 | García Espinosa, Y., et al. (2011). "Maternal nutrition during pregnancy. Risk factor for prematurity and low birth weight." Annals of Nutrition and Metabolism 58: 261. | Type of article was Conference Abstract |
| 37 | Liu, Z., et al. (2011). "Effect of milk and calcium supplementation on bone density and bone turnover in pregnant Chinese women: a randomized controlled trail." Arch Gynecol Obstet 283(2): 205-211. | The outcome was bone density and bone turnover in pregnant women |
| 38 | Myhre, R., et al. (2011). "Intake of probiotic food and risk of spontaneous preterm delivery." Am J Clin Nutr 93(1): 151-157. | The exposure examined was milk-based probiotic products |
| 39 | Yang, Z. and S. L. Huffman (2011). "Review of fortified food and beverage products for pregnant and lactating women and their impact on nutritional status." Matern Child Nutr 7 Suppl 3: 19-43. | Type of article was Review; The exposure was fortified food |
| 40 | Brantsaeter, A. L., et al. (2012). "Does milk and dairy consumption during pregnancy influence fetal growth and infant birthweight? A systematic literature review." Food Nutr Res 56. | Type of article was Review |
| 41 | Chatzi, L., et al. (2012). "Mediterranean diet adherence during pregnancy and fetal growth: INMA (Spain) and RHEA (Greece) mother-child cohort studies." Br J Nutr 107(1): 135-145. | The exposure examined was Mediterranean diet adherence during pregnancy |
| 42 | Nwaru, B. I., et al. (2012). "Maternal intake of fatty acids during pregnancy and allergies in the offspring." Br J Nutr 108(4): 720-732. | The outcome examined was allergies in children aged 5 years |
| 43 | Tanaka, K., et al. (2012). "Dairy products and calcium intake during pregnancy and dental caries in children." Nutr J 11: 33. | The outcome examined was dental caries in 41-50 months children |
| 44 | Anjum, N., et al. (2013). "Role of nutrition in pregnancy and its effect on fetal birth weight." Pakistan Journal of Medical and Health Sciences 7(2). | The exposure examined was nutritional supplement |
| 45 | Borazjani, F., et al. (2013). "Milk and protein intake by pregnant women affects growth of foetus." J Health Popul Nutr 31(4): 435-445. | The outcome examined was foetal measurements |
| 46 | Ganpule-Rao, A., et al. (2013). "Maternal milk calcium consumption improves birth size in rural India." Annals of Nutrition and Metabolism 63: 756. | Type of article was Conference Abstract |
| 47 | Harris, H. R., et al. (2013). "Dairy-food, calcium, magnesium, and vitamin D intake and endometriosis: a prospective cohort study." Am J Epidemiol 177(5): 420-430. | The outcome examined was endometriosis |
| 48 | Hrolfsdottir, L., et al. (2013). "Maternal milk consumption, birth size and adult height of offspring: A prospective cohort study with 20 years of follow-up." Annals of Nutrition and Metabolism 63: 788. | Type of article was Conference Abstract |
| 49 | Brantsæter, A. L., et al. (2014). "Diet matters, particularly in pregnancy – Results from MoBa studies of maternal diet and pregnancy outcomes." Norsk Epidemiologi 24(1-2): 63-77. | It was a summary of the results from 19 studies of maternal diet and pregnancy outcomes based on the MoBa FFQ and published before September 2014. |
| 50 | Bunyavanich, S., et al. (2014). "Peanut, milk, and wheat intake during pregnancy is associated with reduced allergy and asthma in children." Journal of Allergy and Clinical Immunology 133(5): 1373-1382. | The outcome was childhood allergy and asthma |
| 51 | Chen, L.-W., et al. (2014). "Dietary changes during pregnancy and the postpartum period in Singaporean Chinese, Malay and Indian women: the GUSTO birth cohort study." Public Health Nutr 17(9): 1930-1938. | No birth outcomes involved |
| 52 | Cuervo, M., et al. (2014). "Dietary and Health Profiles of Spanish Women in Preconception, Pregnancy and Lactation." Nutrients 6(10): 4434-4451. | No birth outcomes involved |
| 53 | Grieger, J. A. and V. L. Clifton (2014). "A review of the impact of dietary intakes in human pregnancy on infant birthweight." Nutrients 7(1): 153-178. | Type of article was Review |
| 54 | Jensen, C. B., et al. (2014). "Does prenatal exposure to vitamin D-fortified margarine and milk alter birth weight? A societal experiment." British Journal of Nutrition 112(5): 785-793. | The exposure examined was vitamin D fortification |
| 55 | Kjollesdal, M. K. R. and G. Holmboe-Ottesen (2014). "Dietary Patterns and Birth Weight-a Review." AIMS Public Health 1(4): 211-225. | Type of article was Review |
| 56 | Malhotra, N., R. P. Upadhyay, M. Bhilwar, N. Choy and T. Green (2014). "The role of maternal diet and iron-folic acid supplements in influencing birth weight: evidence from India's National Family Health Survey." J Trop Pediatr 60(6): 454-460. | It did not state that the time of exposure (dairy intake) was during pregnancy |
| 57 | Miyake, Y., et al. (2014). "Maternal consumption of dairy products, calcium, and vitamin D during pregnancy and infantile allergic disorders." Ann Allergy Asthma Immunol 113(1): 82-87. | The outcome examined was allergic disorders in children aged 23-29 months |
| 58 | Stephens, T. V., et al. (2014). "Healthy pregnant women in Canada are consuming more dietary protein at 16- and 36-week gestation than currently recommended by the Dietary Reference Intakes, primarily from dairy food sources." Nutr Res 34(7): 569-576. | The exposure was macronutrients and dairy protein |
| 59 | Torjusen, H., et al. (2014). "Reduced risk of pre-eclampsia with organic vegetable consumption: results from the prospective Norwegian Mother and Child Cohort Study." BMJ Open 4(9). | The outcome examined was pre-eclampsia |
| 60 | Coelho Nde, L., et al. (2015). "Dietary patterns in pregnancy and birth weight." Rev Saude Publica 49: 62. | The exposure was dietary patterns |
| 61 | Melnik, B. C., et al. (2015). "Milk consumption during pregnancy increases birth weight, a risk factor for the development of diseases of civilization." J Transl Med 13: 13. | Type of article was Review |
| 62 | Pedersen, M., et al. (2015). "Environmental, dietary, maternal, and fetal predictors of bulky DNA adducts in cord blood: a European mother-child study (NewGeneris)." Environ Health Perspect 123(4): 374-380. | The outcome was bulky DNA adducts |
| 63 | Przybylowicz, K., et al. (2015). "Associations of milk and dairy consumption during pregnancy with infant birth weight." Annals of Nutrition and Metabolism 67: 318-319. | Type of article was Meeting Abstract |
| 64 | Villar-Vidal, M., et al. (2015). "Compliance of nutritional recommendations of Spanish pregnant women according to sociodemographic and lifestyle characteristics: a cohort study." Nutr Hosp 31(4): 1803-1812. | No birth outcomes involved |
| 65 | Xie, Y., et al. (2015). "Preconception Nutrition, Physical Activity, and Birth Outcomes in Adolescent Girls." J Pediatr Adolesc Gynecol 28(6): 471-476. | The exposure was preconception nutrition |
| 66 | Afeiche, M. C., et al. (2016). "Dairy intake in relation to in vitro fertilization outcomes among women from a fertility clinic." Hum Reprod 31(3): 563-571. | The participants were women undergoing in vitro fertilization;the outcomes were in vitro fertilization outcomes |
| 67 | Bengtson, M.-B., et al. (2016). "Restricted Intake of Dairy Protein Increased the Risk of SGA Among IBD Mothers in a Norwegian Population-Based Mother and Child Cohort." Gastroenterology 150(4): S780-S780. | Type of article was Meeting Abstract |
| 68 | Chen, L. W., et al. (2016). "Associations of Maternal Dietary Patterns during Pregnancy with Offspring Adiposity from Birth Until 54 Months of Age." Nutrients 9(1). | The exposure was dietary patterns |
| 69 | Chen, L. W., et al. (2016). "Maternal Macronutrient Intake during Pregnancy Is Associated with Neonatal Abdominal Adiposity: The Growing Up in Singapore Towards healthy Outcomes (GUSTO) Study." J Nutr 146(8): 1571-1579. | The exposure was maternal macronutrient intake |
| 70 | Chia, A.-R., et al. (2016). "A vegetable, fruit, and white rice dietary pattern during pregnancy is associated with a lower risk of preterm birth and larger birth size in a multiethnic Asian cohort: the Growing Up in Singapore Towards healthy Outcomes (GUSTO) cohort study." American Journal of Clinical Nutrition 104(5): 1416-1423. | The exposure was dietary pattern |
| 71 | Clark, D. C. (2016). "Dairy and Growth, Latest Findings, and Lessons Learned." Food Nutr Bull 37 Suppl 1: S22-28. | Type of article was Review |
| 72 | de Seymour, J., et al. (2016). "Maternal Dietary Patterns and Gestational Diabetes Mellitus in a Multi-Ethnic Asian Cohort: The GUSTO Study." Nutrients 8(9). | The exposure was dietary patterns |
| 73 | DiRienzo, D. (2016). "Research Gaps in the Use of Dairy Ingredients in Food Aid Products." Food Nutr Bull 37 Suppl 1: S51-57. | Type of article was Review |
| 74 | Flores-Quijano, M. E. and S. Heller-Rouassant (2016). "[Pregnancy and breastfeeding]." Gac Med Mex 152 Suppl 1: 6-12. | Type of article was Review (in Spanish) |
| 75 | Irct201604013706N (2016) Effect of probiotic yoghurt on level of blood glucose in obese and overweight pregnant women. | The information from Cochrane Central Register of Controlled Trials; Not an article; The intervention was probiotic yoghurt |
| 76 | Li, H., et al. (2016). "A discriminant analysis prediction model of non-syndromic cleft lip with or without cleft palate based on risk factors." BMC Pregnancy Childbirth 16(1): 368. | The exposure examined was Milk/soymilk |
| 77 | Lu, M.-S., et al. (2016). "Maternal Dietary Patterns and Fetal Growth: A Large Prospective Cohort Study in China." Nutrients 8(5). | The exposure examined was dietary pattern |
| 78 | Madanijah, S., et al. (2016). "Nutritional status of pre-pregnant and pregnant women residing in Bogor district, Indonesia: a cross-sectional dietary and nutrient intake study." Br J Nutr 116 Suppl 1: S57-66. | No birth outcomes involved |
| 79 | Malek, L., et al. (2016). "Adherence to the Australian dietary guidelines during pregnancy: evidence from a national study." Public Health Nutr 19(7): 1155-1163. | No birth outcomes involved |
| 80 | Mani, I., et al. (2016). "Maternal fat and fatty acid intake and birth outcomes in a South Indian population." Int J Epidemiol 45(2): 523-531. | The exposure was fat and fatty acid |
| 81 | Shamim, A. A., et al. (2016). "Pregnant Women Diet Quality and Its Sociodemographic Determinants in Southwestern Bangladesh." Food Nutr Bull 37(1): 14-26. | The exposure was diet diversity score; no birth outcomes involved |
| 82 | Takahashi, F., et al. (2016). "Fermented Food Consumption and Psychological Distress in Pregnant Women: A Nationwide Birth Cohort Study of the Japan Environment and Children's Study." Tohoku Journal of Experimental Medicine 240(4): 309-321. | The outcome was psychological distress |
| 83 | Tuokkola, J., et al. (2016). "Maternal diet during pregnancy and lactation and cow's milk allergy in offspring." 70(5): 554-559. | The outcome was cow's milk allergy |
| 84 | Wennberg, A. L., et al. (2016). "Swedish women's food habits during pregnancy up to six months post-partum: A longitudinal study." Sexual & reproductive healthcare : official journal of the Swedish Association of Midwives 8: 31-36. | No birth outcomes involved |
| 85 | Wiley, A. S., et al. (2016). "Cord IGF-I concentrations in Indian newborns: associations with neonatal body composition and maternal determinants." Pediatr Obes 11(2): 151-157. | The outcome was cord IGF-I |
| 86 | Amini, S., et al. (2017). "Comparison of food intake and body mass index before pregnancy between women with spontaneous abortion and women with successful pregnancy." Iranian journal of obstetrics, gynecology and infertility 20(10): 35-42. | The full-text was in Persian |
| 87 | Dąbrowski, M. and D. Pawluś (2017). "Association of food choices during pregnancy with gestational diabetes mellitus." Clinical Diabetology 6(4): 131-135. | The outcome was gestational diabetes mellitus |
| 88 | Eshriqui, I., et al. (2017). "Prepregnancy Dietary Patterns Are Associated with Blood Lipid Level Changes During Pregnancy: A Prospective Cohort Study in Rio de Janeiro, Brazil." J Acad Nutr Diet 117(7): 1066-1079. | The exposure was prepregnancy dietary patterns; no birth oucomes involved |
| 89 | Hiersch, L. and Y. Yogev (2017). Pregnancy: Impact of Maternal Nutrition on Intrauterine Fetal Growth. Nutrition and Growth: Yearbook 2017. B. Koletzko, R. Shamir, D. Turck and M. Phillip. 116: 152-164. | It was a book chapter |
| 90 | Liu, X., et al. (2017). "Reduced maternal calcium intake through nutrition and supplementation is associated with adverse conditions for both the women and their infants in a Chinese population." Medicine (Baltimore) 96(18): e6609. | No birth outcomes involved |
| 91 | Loo, E. X. L., et al. (2017). "Effect of Maternal Dietary Patterns during Pregnancy on Self-Reported Allergic Diseases in the First 3 Years of Life: Results from the GUSTO Study." International Archives of Allergy and Immunology 173(2): 105-113. | The exposure was dietary pattern; the outcome was allergic disease |
| 92 | Al Hilali, M., et al. (2018). "Periconceptional maternal dairy-rich dietary pattern is associated with prenatal cerebellar growth." PLoS One 13(5): e0197901. | The exposure was periconceptional dietary pattern |
| 93 | Bartáková, V., et al. (2018). "Differences in food intake and genetic variability in taste receptors between Czech pregnant women with and without gestational diabetes mellitus." Eur J Nutr 57(2): 513-521. | The outcome was gestational diabetes mellitus |
| 94 | Clark, D. C. (2018). "Association of Dairy Protein Intake During Pregnancy with Birth Weight." Food Nutr Bull 39(2_suppl): S54-s59. | Type of article was Review |
| 95 | Collins, C. T., et al. (2018). "Adherence to the New Nordic Diet during pregnancy and subsequent maternal weight development: a study conducted in the Norwegian Mother and Child Cohort Study (MoBa)." Nutrients 119(11): 1286-1294. | The exposure was new Nordic dietary pattern |
| 96 | Forbes, L. E., et al. (2018). "Dietary Change during Pregnancy and Women's Reasons for Change." Nutrients 10(8). | No birth outcomes involved |
| 97 | Gaskins, A. J. and J. E. Chavarro (2018). "Diet and fertility: a review." Am J Obstet Gynecol 218(4): 379-389. | Type of article was Review; the outcome was fetility |
| 98 | Hrolfsdottir, L. (2018). "Development of a dietary screening questionnaire to predict excessive weight gain in pregnancy." Nutr J: e12639. | The outcome was gestational weight gain |
| 99 | Liang, Y., et al. (2018). "Dietary Protein Intake, Meat Consumption, and Dairy Consumption in the Year Preceding Pregnancy and During Pregnancy and Their Associations With the Risk of Gestational Diabetes Mellitus: A Prospective Cohort Study in Southwest China." Front Endocrinol (Lausanne) 9: 596. | The outcome was Gestational Diabetes Mellitus |
| 100 | Louvigne, M., et al. (2018). "Association of maternal nutrition with transient neonatal hyperinsulinism." PLoS One 13(5). | The outcome was transient neonatal hyperinsulinism |
| 101 | Nordqvist, M., et al. (2018). "Timing of probiotic milk consumption during pregnancy and effects on the incidence of preeclampsia and preterm delivery: a prospective observational cohort study in Norway." BMJ Open 8(1): e018021. | The exposure was probiotic milk |
| 102 | Lu, M. S., et al. (2018). "Maternal dietary patterns during pregnancy and preterm delivery: a large prospective cohort study in China." 17(1): 71. | The exposure was dietary pattern |
| 103 | Saeed, A., et al. (2018). "Maternal dietary intake and its associated socio demographic factors: Evidence from Lahore, Pakistan." Progress in Nutrition 20: 345-351. | No birth outcomes involved |
| 104 | Zaidi, T. H., et al. (2018). "Frequency of low birth weight babies and Scio-economic status among the mothers of Karachi." Medical Forum Monthly 29(12): 2-5. | A descriptive cross-sectional study without control group |
| 105 | Achon, M., et al. (2019). "Effects of Milk and Dairy Product Consumption on Pregnancy and Lactation Outcomes: A Systematic Review." Adv Nutr 10(suppl_2): S74-s87. | Type of article was Review |
| 106 | Alves-Santos, N. H., et al. (2019). "Prepregnancy Dietary Patterns and Their Association with Perinatal Outcomes: A Prospective Cohort Study." J Acad Nutr Diet. | The exposure was prepregnancy dietary patterns |
| 107 | Aubert, A. M., et al. (2019). "Deriving the Dietary Approaches to Stop Hypertension (DASH) Score in Women from Seven Pregnancy Cohorts from the European ALPHABET Consortium." Nutrients 11(11). | No birth outcomes involved |
| 108 | Bosha, T., et al. (2019). "Dietary diversity and anthropometric status of mother–child pairs from enset (False banana) staple areas: A panel evidence from southern Ethiopia." Int J Environ Res Public Health 16(12). | No birth outcomes involved |
| 109 | Chia, A.-R., et al. (2019). "Maternal Dietary Patterns and Birth Outcomes: A Systematic Review and Meta-Analysis." Adv Nutr. | Type of article was Review |
| 110 | Gil, A. and R. M. Ortega (2019). "Introduction and Executive Summary of the Supplement, Role of Milk and Dairy Products in Health and Prevention of Noncommunicable Chronic Diseases: A Series of Systematic Reviews." Adv Nutr 10(suppl_2): S67-s73. | Type of article was Review |
| 111 | Hrolfsdottir, L., et al. (2019). "Development of a dietary screening questionnaire to predict excessive weight gain in pregnancy." Maternal and Child Nutrition 15(1). | The outcome was maternal weight gain |
| 112 | Iqbal, S., et al. (2019). "Iron and Iodine Status in Pregnant Women from A Developing Country and Its Relation to Pregnancy Outcomes." Int J Environ Res Public Health 16(22). | The outcome was maternal iron and iodine status |
| 113 | Lai, J. S., et al. (2019). "Macronutrient composition and food groups associated with gestational weight gain: the GUSTO study." Eur J Nutr 58(3): 1081-1094. | The outcome was gestational weight gain |
| 114 | Marangoni, F., et al. (2019). "Cow’s Milk Consumption and Health: A Health Professional’s Guide." J Am Coll Nutr 38(3): 197-208. | Type of article was Review |
| 115 | Munda, A., et al. (2019). "Association between pre-pregnancy body weight and dietary pattern with large-for-gestational-age infants in gestational diabetes." Diabetology & Metabolic Syndrome 11(1). | The outcome was maternal weight gain |
| 116 | Sahhaf Ebrahimi, F., et al. (2019). "Effect of L. acidophilus and B. lactis on blood glucose in women with gestational diabetes mellitus: a randomized placebo-controlled trial." Diabetology & Metabolic Syndrome 11(1). | The exoposure was probiotic yoghurt |
| 117 | Tyagi, S., et al. (2019). "Maternal Dietary Intake During Pregnancy and Its Association with Size of Offspring at Birth and One Year of Age (P11-031-19)." Current developments in nutrition 3(Suppl 1). | Type of article was Abstract |
| 118 | Wei, X., et al. (2019). "The influence of maternal dietary patterns on gestational weight gain: A large prospective cohort study in China." Nutrition 59: 90-95. | The exposure was dietary patterns; the outcome was gestational weight gain |
| 119 | Welderufael, A. L., et al. (2019). "Nutritional status among women whose pregnancy outcome was afflicted with neural tube defects in Tigray region of Ethiopia." Brain Dev 41(5): 406-412. | Exposure time is not during pregnancy |
| 120 | Adalsteinsdottir, S., et al. (2020). "Insufficient iodine status in pregnant women as a consequence of dietary changes." Food Nutr Res 64. | The outcome was maternal iodine status |
| 121 | Bengtson, M. B., et al. (2020). "Intake of dairy protein during pregnancy in IBD and risk of SGA in a Norwegian population-based mother and child cohort." BMC Gastroenterol 20(1). | The exposure was dairy protein |
| 122 | Bjerregaard, A. A., et al. (2020). "Changes in dietary preferences reported in pregnancy: Associations with later pregnancy complications in a sample of 55,087 women." Proceedings of the Nutrition Society 79(OCE2). | Type of article was Abstract |
| 123 | Cano-Ibáñez, N., et al. (2020). "Maternal dietary diversity and risk of small for gestational age newborn: Findings from a case-control study." Clin Nutr 39(6): 1943-1950. | The exposure was dietary diversity score |
| 124 | Diaz-Lopez, A., et al. (2020). "Prevalence and risk factors of hypovitaminosis D in pregnant Spanish women." Sci Rep 10(1). | The outcome was hypovitaminosis D in pregnant Spanish women |
| 125 | Dineva, M., et al. (2020). "Similarities and differences of dietary and other determinants of iodine status in pregnant women from three European birth cohorts." Eur J Nutr 59(1): 371-387. | The outcome was maternal iodine status |
| 126 | Hirko, K. A., et al. (2020). "Diet during Pregnancy and Gestational Weight Gain in a Michigan Pregnancy Cohort." Curr Dev Nutr 4(8): nzaa121. | The outcome was gestational weight gain |
| 127 | Perreault, M., et al. (2020). "Summer Season and Recommended Vitamin D Intake Support Adequate Vitamin D Status throughout Pregnancy in Healthy Canadian Women and Their Newborns." J Nutr 150(4): 739-746. | The exposure was Vitamin D intake from milk;The outcome was maternal serum 25(OH)D concentrations |
| 128 | Pinho-Pompeu, M., et al. (2020). "Influence of breakfast and meal frequency in calcium intake among pregnant adolescents." Matern Child Nutr 16(4): e13034. | The outcome was calcium intake of pregnant Brazilian adolescents |
| 129 | Rust, P., et al. (2020). "Improving Health of the Next Generation: Dietary Intake and Physical Activity during Pregnancy in an Austrian Cohort." Proceedings of the Nutrition Society 79(OCE2). | Type of article was Abstract |
| 130 | Stravik, M., et al. (2020). "Maternal Intake of Cow's Milk during Lactation Is Associated with Lower Prevalence of Food Allergy in Offspring." Nutrients 12(12). | The outcome was physician-diagnosed allergy in the offspring at 12 months of age. |
| 131 | Abu-Saad, K., et al. (2021). "Preconceptional diet quality is associated with birth outcomes among low socioeconomic status minority women in a high-income country." Eur J Nutr 60(1): 65-77. | The exposure was preconceptional diet quality |
| 132 | Addissie, Y. A., et al. (2021). "Identifying environmental risk factors and gene-environment interactions in holoprosencephaly." Birth Defects Research 113(1): 63-76. | The frequency of cheese consumption was per month during the 3 months before pregnancy and during pregnancy |
| 133 | Halldorsson, T. I., et al. (2021). "Old question revisited: Are high-protein diets safe in pregnancy?" Nutrients 13(2): 1-12. | The exposure was protein |
| 134 | Threapleton, D. E., et al. (2021). "Prenatal and Postpartum Maternal Iodide Intake from Diet and Supplements, Urinary Iodine and Thyroid Hormone Concentrations in a Region of the United Kingdom with Mild-to-Moderate Iodine Deficiency." Nutrients 13(1). | The outcomes were prenatal and postpartum maternal iodide intake |
