# Supplementary material for: Maternal Consumption of Milk or Dairy Products During Pregnancy and Birth Outcomes: A Systematic Review and Dose-Response Meta-Analysis
Source: Front Nutr. 2022 Jun 9;9:900529. doi: 10.3389/fnut.2022.900529 (PMC9261982; doi:10.3389/fnut.2022.900529)
Supplement: Supplementary file 3 [file Data_Sheet_2.docx]

Supplementary Material

**Supplementary Material 3.** Summary of the quality assessments

**Supplementary** **Table 3.1** Quality assessment of Cohort studies

| **Criteria** | **Rao 2001** | **Chang 2003** | **Mannion 2006** | **Olsen 2007** | **Kanade 2008** | **Heppe 2011** | **Maslova2012** | **Hrolfsdottir 2013** | **Shaikh 2014** | **Akbari 2015** | **Brantsaeter 2016** | **Miyake 2016** |
| --- | --- | --- | --- | --- | --- | --- | --- | --- | --- | --- | --- | --- |
|  |  |  |  |  |  |  |  |  |  |  |  |  |
| 1. Was the research question or objective in this paper clearly stated? | Y | Y | Y | Y | Y | Y | Y | Y | Y | Y | Y | Y |
| 2. Was the study population clearly specified and defined? | Y | Y | Y | Y | Y | Y | Y | Y | Y | Y | Y | Y |
| 3. Was the participation rate of eligible persons at least 50%? | Y | N | N | N | Y | Y | N | Y | NR | NR | N | NR |
| 4. Were all the subjects selected or recruited from the same or similar populations (including the same time period)? Were inclusion and exclusion criteria for being in the study prespecified and applied uniformly to all participants? | Y | Y | Y | Y | Y | Y | Y | Y | Y | Y | Y | Y |
| 5. Was a sample size justification, power description, or variance and effect estimates provided? | NR | NR | NR | NR | NR | NR | NR | NR | NR | NR | NR | NR |
| 6. For the analyses in this paper, were the exposure(s) of interest measured prior to the outcome(s) being measured? | Y | Y | Y | Y | Y | Y | Y | Y | Y | CD | Y | Y |
| 7. Was the timeframe sufficient so that one could reasonably expect to see an association between exposure and outcome if it existed? | Y | Y | Y | Y | Y | Y | Y | Y | Y | CD | Y | Y |
| 8. For exposures that can vary in amount or level, did the study examine different levels of the exposure as related to the outcome (e.g., categories of exposure, or exposure measured as continuous variable)? | Y | Y | Y | Y | Y | Y | Y | Y | Y | Y | N | N |
| 9. Were the exposure measures (independent variables) clearly defined, valid, reliable, and implemented consistently across all study participants? | CD | CD | Y | Y | CD | Y | Y | Y | CD | CD | CD | Y |
| 10. Was the exposure(s) assessed more than once over time? | Y | N | Y | Y | Y | N | N | N | N | N | N | N |
| 11. Were the outcome measures (dependent variables) clearly defined, valid, reliable, and implemented consistently across all study participants? | Y | Y | CD | Y | CD | CD | N | CD | Y | Y | CD | CD |
| 12. Were the outcome assessors blinded to the exposure status of participants? | NR | CD | NR | NR | NR | NR | NR | NR | NR | NR | NR | NR |
| 13. Was loss to follow-up after baseline 20% or less? | N | Y | Y | Y | Y | Y | Y | Y | N | Y | Y | N |
| 14. Were key potential confounding variables measured and adjusted statistically for their impact on the relationship between exposure(s) and outcome(s)? | Y | Y | Y | Y | Y | Y | CD | Y | N | N | Y | N |
| Quality score | 10 | 9 | 10 | 11 | 10 | 10 | 8 | 10 | 7 | 6 | 7 | 6 |

Y,yes; N,no; CD, cannot determine; NA, not applicable; NR, not reported

**Supplementary** **Table 3.1** Quality assessment of Cohort studies (Continued)

| **Criteria** | **Olmedo 2016** | **Abreu 2017** | **Kriss 2018** | **Mukhopadhyay 2018** | **Zerfu 2018** | **Ito 2019** | **Shen 2019** | **Assaf-Balut 2020** | **Pang 2020** | **Sartorelli 2021** | **Voerman 2021** |
| --- | --- | --- | --- | --- | --- | --- | --- | --- | --- | --- | --- |
|  |  |  |  |  |  |  |  |  |  |  |  |
| 1. Was the research question or objective in this paper clearly stated? | Y | Y | Y | Y | Y | Y | Y | Y | Y | Y | Y |
| 2. Was the study population clearly specified and defined? | Y | Y | Y | CD | CD | Y | Y | Y | Y | Y | Y |
| 3. Was the participation rate of eligible persons at least 50%? | N | Y | Y | NR | NR | Y | NR | Y | CD | Y | CD |
| 4. Were all the subjects selected or recruited from the same or similar populations (including the same time period)? Were inclusion and exclusion criteria for being in the study prespecified and applied uniformly to all participants? | Y | Y | N | Y | Y | Y | Y | CD | Y | Y | Y |
| 5. Was a sample size justification, power description, or variance and effect estimates provided? | NR | Y | Y | NR | Y | NR | NR | NR | NR | Y | NR |
| 6. For the analyses in this paper, were the exposure(s) of interest measured prior to the outcome(s) being measured? | Y | Y | Y | Y | Y | Y | Y | Y | Y | Y | Y |
| 7. Was the timeframe sufficient so that one could reasonably expect to see an association between exposure and outcome if it existed? | Y | Y | Y | Y | Y | Y | Y | Y | Y | Y | Y |
| 8. For exposures that can vary in amount or level, did the study examine different levels of the exposure as related to the outcome (e.g., categories of exposure, or exposure measured as continuous variable)? | Y | Y | Y | Y | N | Y | Y | Y | Y | N | Y |
| 9. Were the exposure measures (independent variables) clearly defined, valid, reliable, and implemented consistently across all study participants? | Y | CD | CD | Y | CD | CD | NR | CD | CD | CD | Y |
| 10. Was the exposure(s) assessed more than once over time? | N | Y | N | Y | Y | Y | N | N | Y | Y | N |
| 11. Were the outcome measures (dependent variables) clearly defined, valid, reliable, and implemented consistently across all study participants? | CD | Y | Y | Y | CD | CD | CD | CD | Y | Y | N |
| 12. Were the outcome assessors blinded to the exposure status of participants? | NR | NR | NR | NR | NR | NR | NR | NR | NR | NR | CD |
| 13. Was loss to follow-up after baseline 20% or less? | N | N | Y | Y | Y | Y | CD | CD | Y | Y | N |
| 14. Were key potential confounding variables measured and adjusted statistically for their impact on the relationship between exposure(s) and outcome(s)? | Y | Y | Y | Y | Y | Y | Y | N | Y | Y | N |
| Quality score | 8 | 11 | 10 | 10 | 8 | 10 | 7 | 6 | 10 | 11 | 7 |

Y,yes; N,no; CD, cannot determine; NA, not applicable; NR, not reported

**Supplementary** **Table 3.2** Quality assessment of Case-control studies

| **Criteria** | **Di Cintio 2001** | **Mitchell 2004** | **Christensen 2013** | **Wang 2015** | **Ahmadi 2017** | **Olmedo-Requena 2019** | **Li 2021** |
| --- | --- | --- | --- | --- | --- | --- | --- |
|  |  |  |  |  |  |  |  |
| 1. Was the research question or objective in this paper clearly stated and appropriate? | Y | Y | Y | Y | Y | Y | Y |
| 2. Was the study population clearly specified and defined? | Y | Y | Y | Y | CD | Y | Y |
| 3. Did the authors include a sample size justification? | NR | Y | CD | NR | Y | Y | NR |
| 4. Were controls selected or recruited from the same or similar population that gave rise to the cases (including the same timeframe)? | Y | Y | Y | Y | Y | Y | Y |
| 5. Were the definitions, inclusion and exclusion criteria, algorithms or processes used to identify or select cases and controls valid, reliable, and implemented consistently across all study participants? | NR | Y | N | CD | Y | Y | Y |
| 6. Were the cases clearly defined and differentiated from controls? | Y | Y | Y | Y | Y | Y | Y |
| 7. If less than 100 percent of eligible cases and/or controls were selected for the study, were the cases and/or controls randomly selected from those eligible? | CD | Y | N | N | CD | N | NR |
| 8. Was there use of concurrent controls? | Y | NR | Y | Y | Y | Y | Y |
| 9. Were the investigators able to confirm that the exposure/risk occurred prior to the development of the condition or event that defined a participant as a case? | N | N | N | N | N | N | N |
| 10. Were the measures of exposure/risk clearly defined, valid, reliable, and implemented consistently (including the same time period) across all study participants? | Y | CD | CD | Y | Y | Y | CD |
| 11. Were the assessors of exposure/risk blinded to the case or control status of participants? | NR | CD | NR | NR | NR | NR | NR |
| 12. Were key potential confounding variables measured and adjusted statistically in the analyses? If matching was used, did the investigators account for matching during study analysis? | Y | Y | CD | Y | N | CD | Y |
| Quality score | 7 | 8 | 5 | 7 | 7 | 8 | 7 |

Y,yes; N,no; CD, cannot determine; NA, not applicable; NR, not reported

**Supplementary** **Table 3.3** Quality assessment of Cross-sectional studies

| **Criteria** | **Petridou 1998** | **Ludvigsson 2004** | **Xue 2008** | **Borazjani 2011** | **Sultan Azzeh 2013** | **Yan 2017** | **Hjertholm 2018** | **Abera 2019** | **Yildirim 2019** | **Rodrigues 2020** |
| --- | --- | --- | --- | --- | --- | --- | --- | --- | --- | --- |
|  |  |  |  |  |  |  |  |  |  |  |
| 1. Was the research question or objective in this paper clearly stated? | Y | Y | Y | Y | Y | Y | Y | Y | Y | Y |
| 2. Was the study population clearly specified and defined? | Y | Y | Y | Y | N | Y | Y | Y | Y | Y |
| 3. Was the participation rate of eligible persons at least 50%? | Y | Y | Y | NR | NR | Y | Y | N | NR | NR |
| 4. Were all the subjects selected or recruited from the same or similar populations (including the same time period)? Were inclusion and exclusion criteria for being in the study prespecified and applied uniformly to all participants? | Y | Y | Y | Y | Y | Y | Y | Y | Y | Y |
| 5. Was a sample size justification, power description, or variance and effect estimates provided? | CD | NR | NR | NR | NR | NR | Y | Y | NR | Y |
| 6. For the analyses in this paper, were the exposure(s) of interest measured prior to the outcome(s) being measured? | N | N | N | N | N | N | Y | N | N | N |
| 7. Was the timeframe sufficient so that one could reasonably expect to see an association between exposure and outcome if it existed? | N | N | N | N | N | N | N | N | N | N |
| 8. For exposures that can vary in amount or level, did the study examine different levels of the exposure as related to the outcome (e.g., categories of exposure, or exposure measured as continuous variable)? | Y | Y | Y | Y | N | Y | Y | Y | CD | Y |
| 9. Were the exposure measures (independent variables) clearly defined, valid, reliable, and implemented consistently across all study participants? | Y | CD | NR | CD | N | CD | CD | CD | CD | Y |
| 10. Was the exposure(s) assessed more than once over time? | N | N | N | N | N | N | N | N | N | N |
| 11. Were the outcome measures (dependent variables) clearly defined, valid, reliable, and implemented consistently across all study participants? | CD | CD | N | CD | CD | CD | Y | Y | Y | Y |
| 12. Were the outcome assessors blinded to the exposure status of participants? | NR | CD | NR | NR | NR | NR | NR | NR | NR | NR |
| 13. Was loss to follow-up after baseline 20% or less? | NA | NA | NA | NA | NA | NA | N | NA | NA | NA |
| 14. Were key potential confounding variables measured and adjusted statistically for their impact on the relationship between exposure(s) and outcome(s)? | Y | Y | Y | Y | Y | Y | Y | Y | Y | Y |
| Quality score | 7 | 6 | 6 | 5 | 3 | 6 | 9 | 7 | 5 | 8 |

Y,yes; N,no; CD, cannot determine; NA, not applicable; NR, not reported

**Supplementary** **Table 3.4** Quality assessment of Interventional studies

| **Criteria** | **Chan 2006** | **Li 2014** |
| --- | --- | --- |
|  |  |  |
| 1. Was the study described as randomized, a randomized trial, a randomized clinical trial, or an RCT? | Y | Y |
| 2. Was the method of randomization adequate (i.e., use of randomly generated assignment)? | Y | N |
| 3. Was the treatment allocation concealed (so that assignments could not be predicted)? | Y | NR |
| 4. Were study participants and providers blinded to treatment group assignment? | NR | NR |
| 5. Were the people assessing the outcomes blinded to the participants' group assignments? | NR | NR |
| 6. Were the groups similar at baseline on important characteristics that could affect outcomes (e.g., demographics, risk factors, co-morbid conditions)? | Y | CD |
| 7. Was the overall drop-out rate from the study at endpoint 20% or lower of the number allocated to treatment? | Y | N |
| 8. Was the differential drop-out rate (between treatment groups) at endpoint 15 percentage points or lower? | Y | NR |
| 9. Was there high adherence to the intervention protocols for each treatment group? | N | NR |
| 10. Were other interventions avoided or similar in the groups (e.g., similar background treatments)? | Y | Y |
| 11. Were outcomes assessed using valid and reliable measures, implemented consistently across all study participants? | CD | CD |
| 12. Did the authors report that the sample size was sufficiently large to be able to detect a difference in the main outcome between groups with at least 80% power? | Y | NR |
| 13. Were outcomes reported or subgroups analyzed prespecified (i.e., identified before analyses were conducted)? | Y | Y |
| 14. Were all randomized participants analyzed in the group to which they were originally assigned, i.e., did they use an intention-to-treat analysis? | Y | N |
| Quality score | 10 | 3 |
| Y,yes; N,no; CD, cannot determine; NA, not applicable; NR, not reported | | |
