# Supplementary material for: Maternal Consumption of Milk or Dairy Products During Pregnancy and Birth Outcomes: A Systematic Review and Dose-Response Meta-Analysis
Source: Front Nutr. 2022 Jun 9;9:900529. doi: 10.3389/fnut.2022.900529 (PMC9261982; doi:10.3389/fnut.2022.900529)
Supplement: Supplementary file 4 [file Data_Sheet_3.docx]

Supplementary Material

# Supplementary Material 4. Supplementary Figures

**
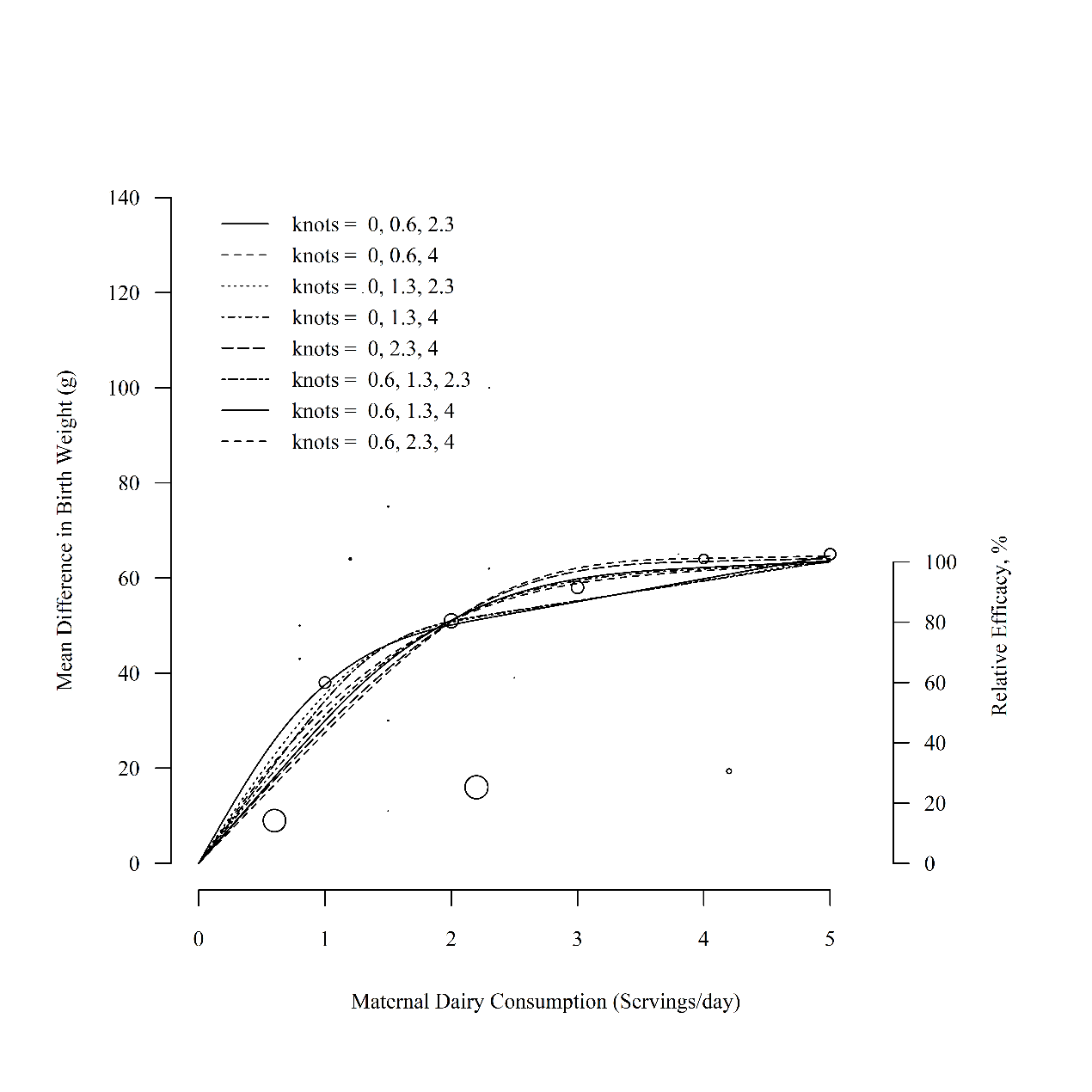
**

**Fig. S1** Sensitivity analysis for pooled dose-response curves between dairy consumption and mean change in birth weight.


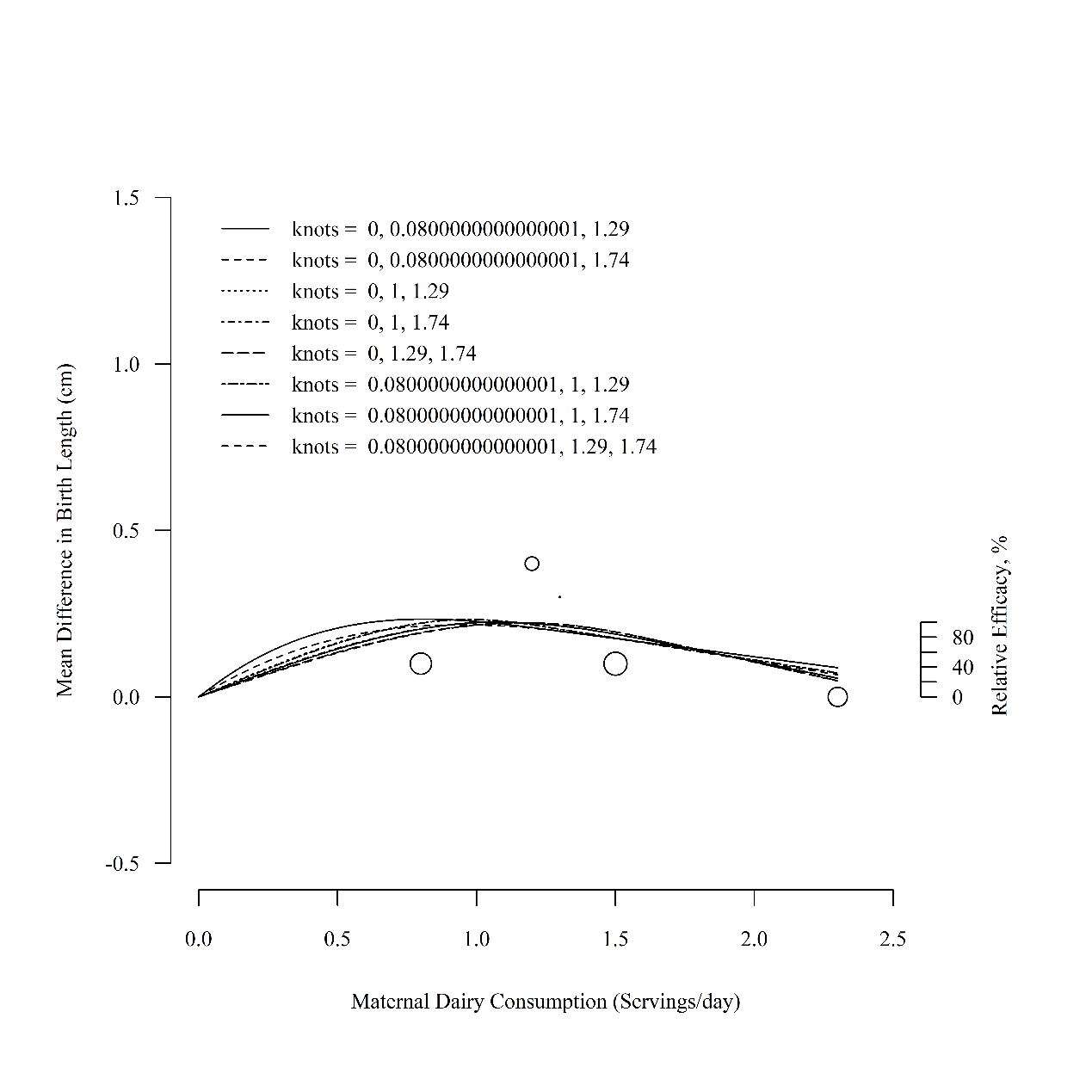


**Fig. S2** Sensitivity analysis for pooled dose-response curves between dairy consumption and mean change in birth length.


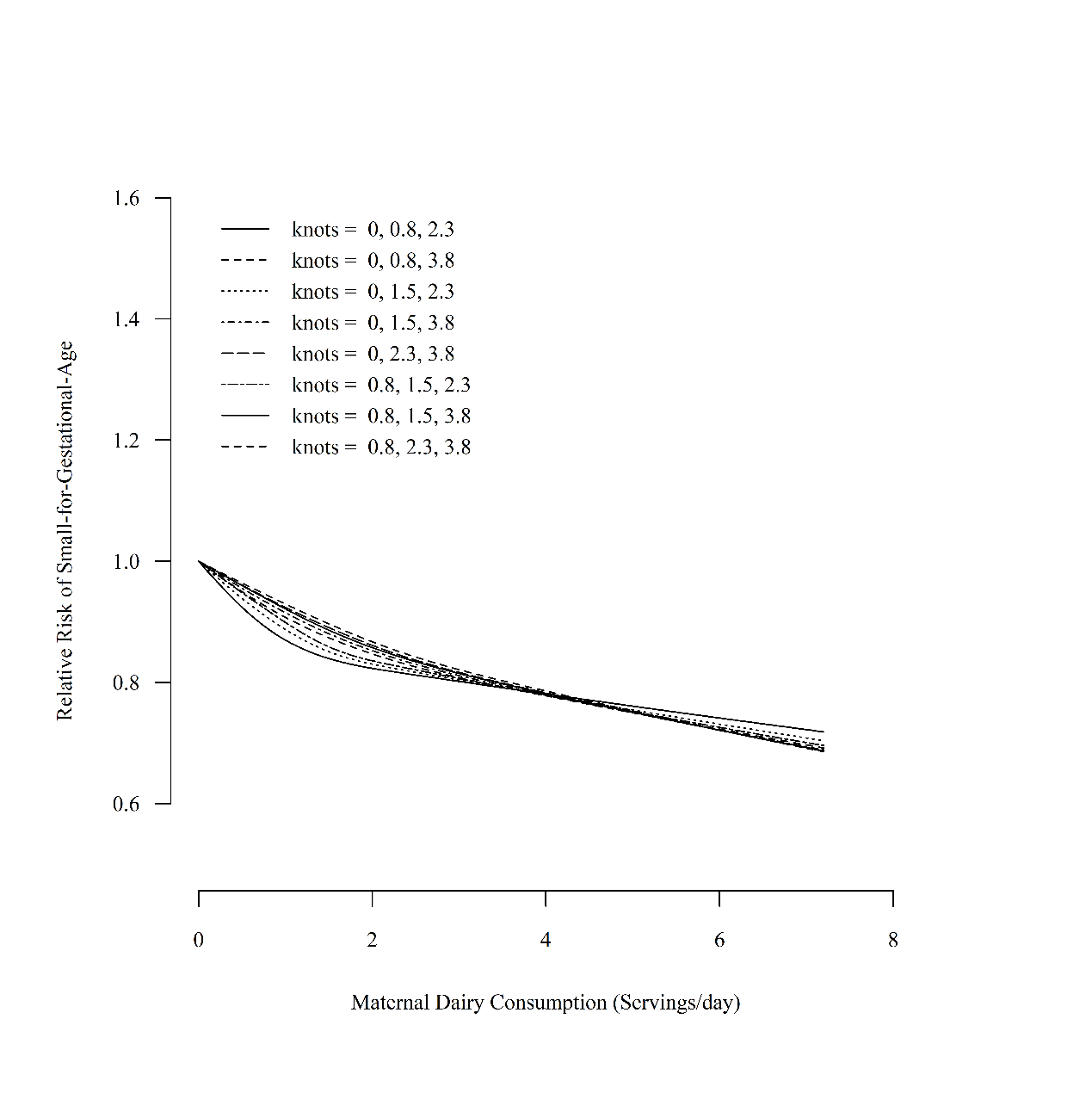


**Fig. S3** Sensitivity analysis for pooled dose-response curves between dairy consumption and the risk of small for gestational age.


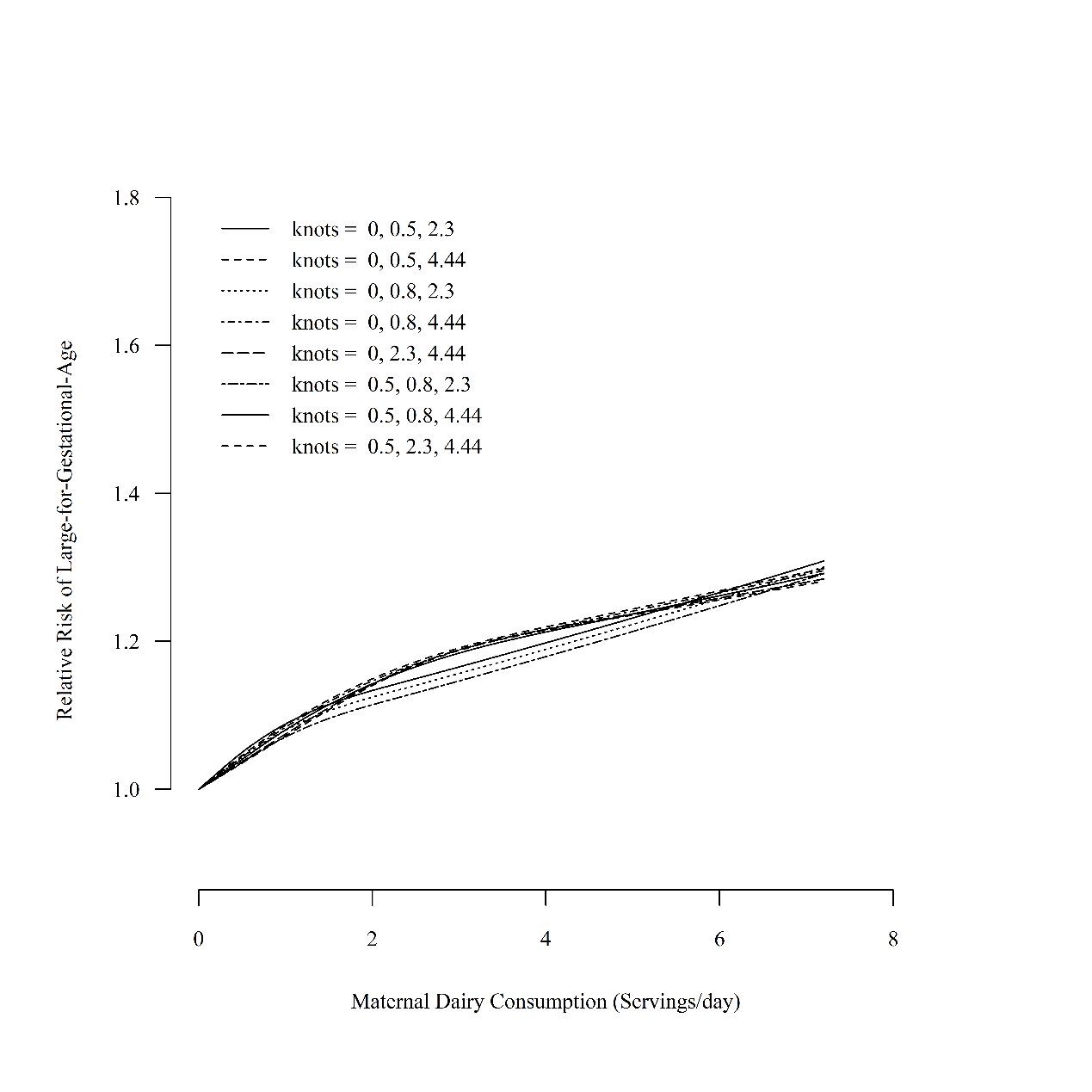


**Fig. S4** Sensitivity analysis for pooled dose-response curves between dairy consumption and the risk of large for gestational age.


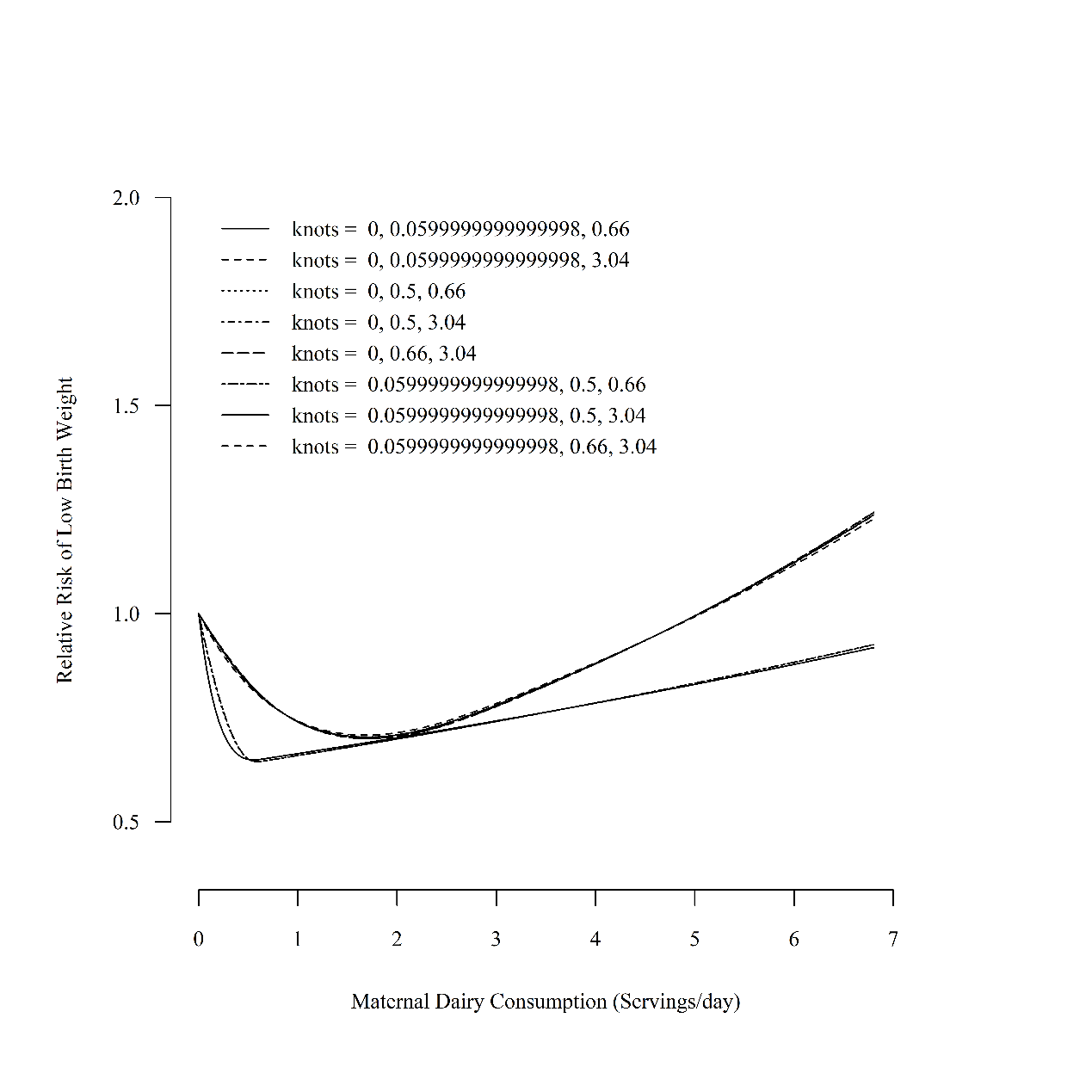


**Fig. S5** Sensitivity analysis for pooled dose-response curves between dairy consumption and the risk of low birth weight.


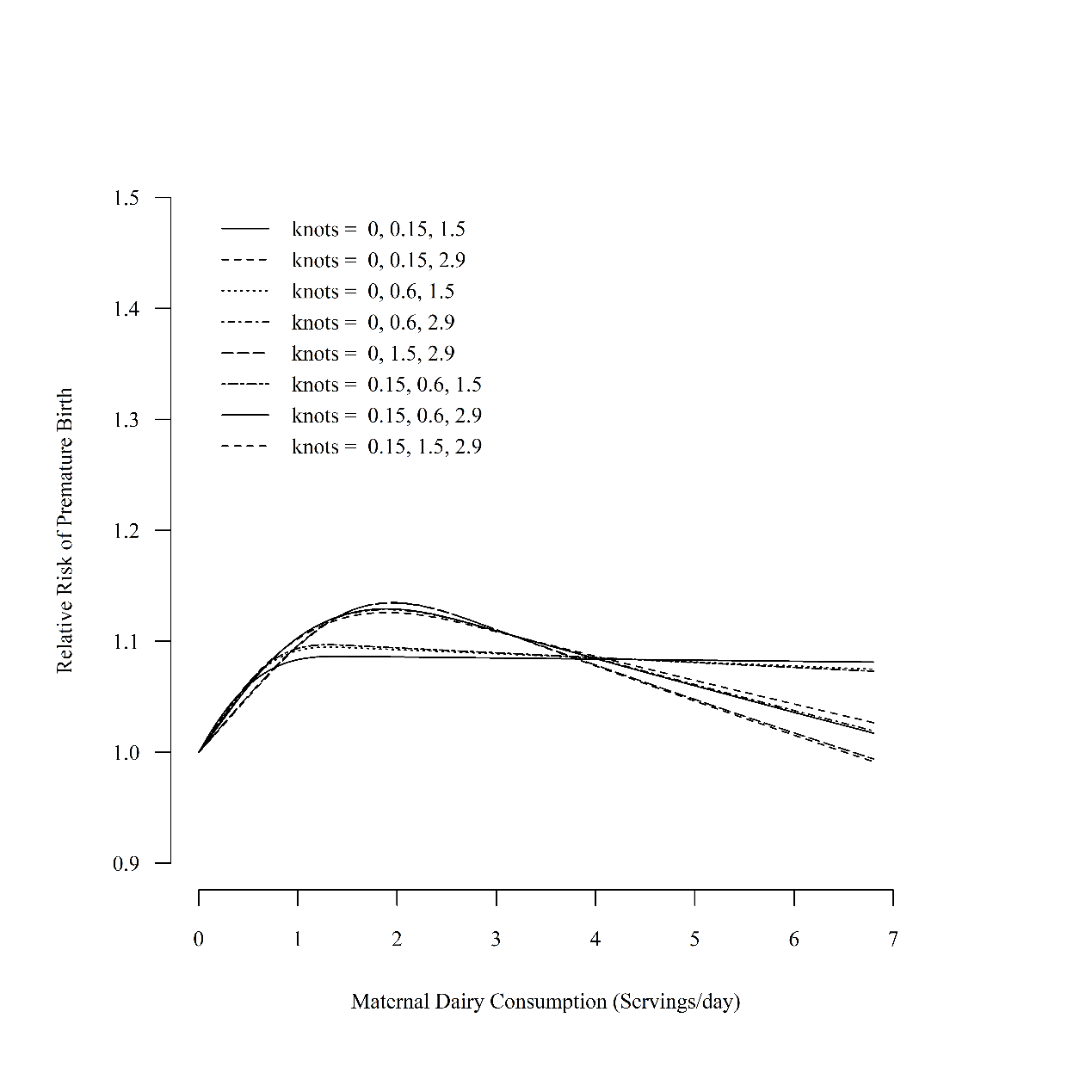


**Fig. S6** Sensitivity analysis for pooled dose-response curves between dairy consumption and the risk of premature birth.


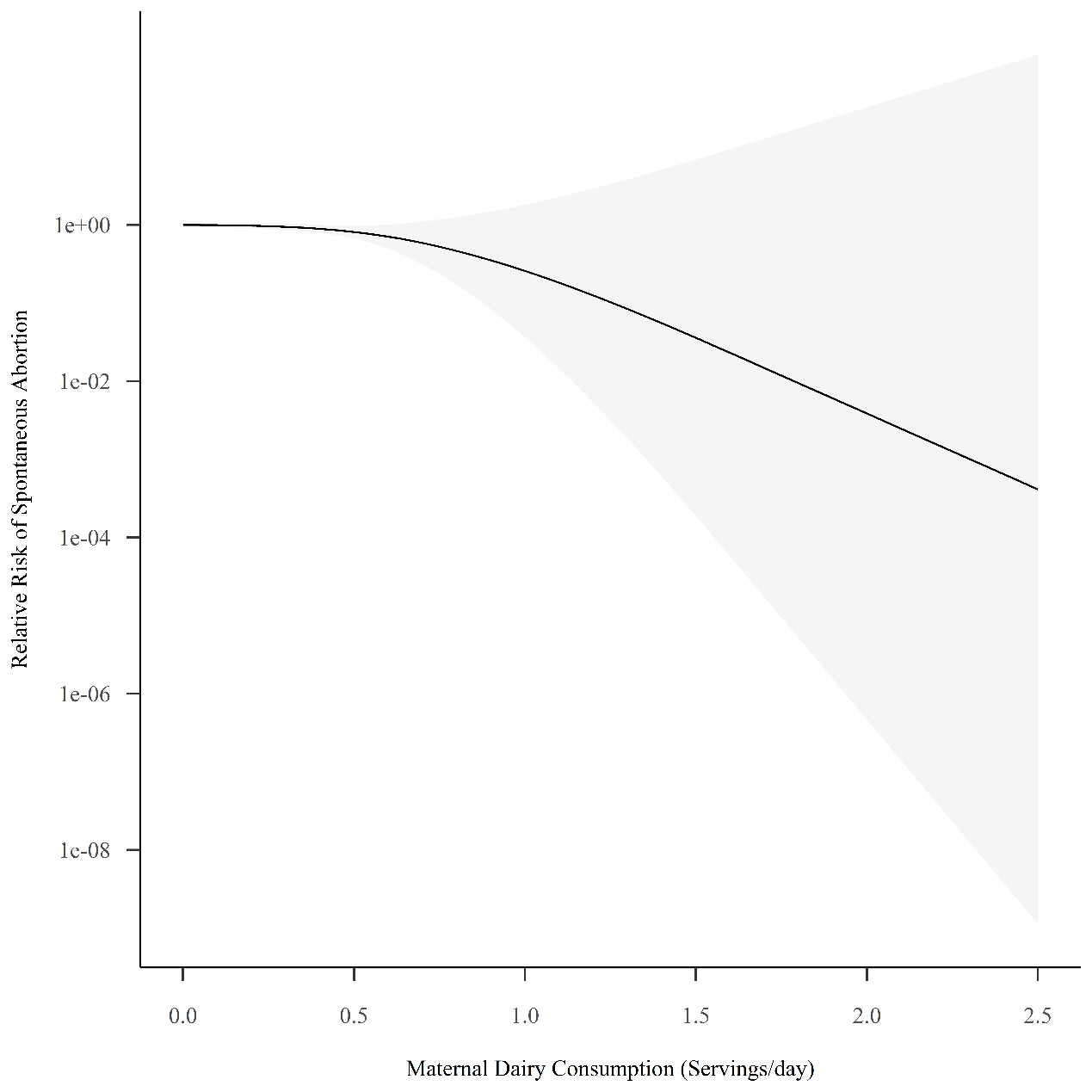


**Fig. S7** Pooled dose-response curves for the risk of spontaneous abortion with maternal dairy consumption.


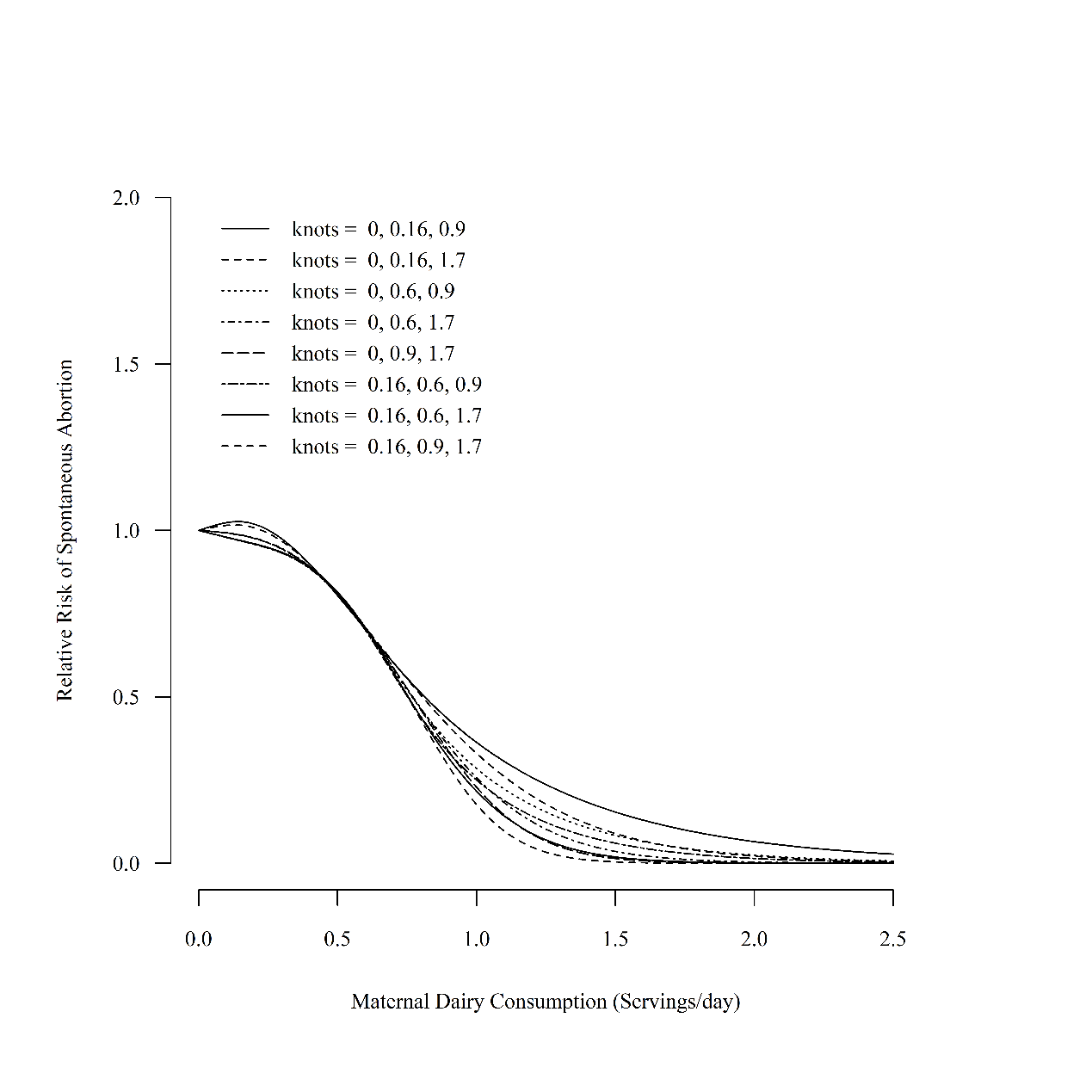


**Fig. S8** Sensitivity analysis for pooled dose-response curves between dairy consumption and the risk of spontaneous abortion.


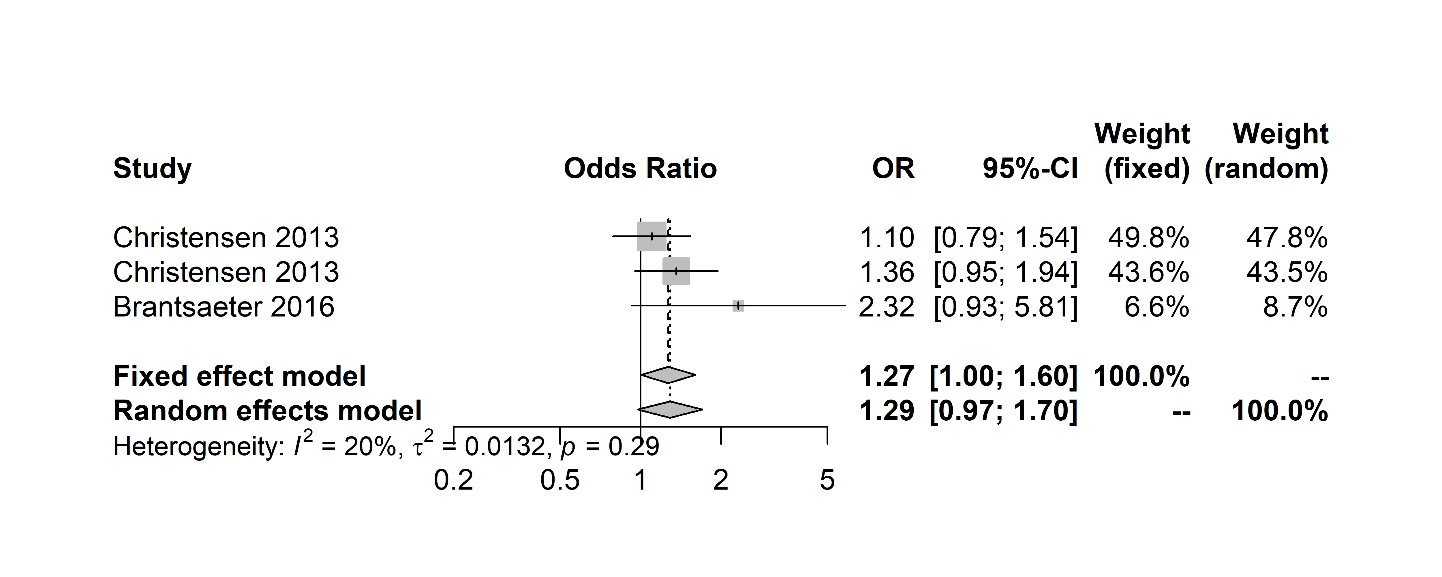


**Fig. S9** The forest plot for the risk of hypospadias with organic dairy consumption during pregnancy.
